# Cell types of origin of the cell-free transcriptome

**DOI:** 10.1038/s41587-021-01188-9

**Published:** 2022-02-07

**Authors:** Sevahn K. Vorperian, Mira N. Moufarrej, Tabula Sapiens Consortium, Robert C. Jones, Jim Karkanias, Mark Krasnow, Angela Oliveira Pisco, Stephen R. Quake, Julia Salzman, Nir Yosef, Bryan Bulthaup, Phillip Brown, William Harper, Marisa Hemenez, Ravikumar Ponnusamy, Ahmad Salehi, Bhavani A. Sanagavarapu, Eileen Spallino, Ksenia A. Aaron, Waldo Concepcion, James M. Gardner, Burnett Kelly, Nikole Neidlinger, Zifa Wang, Sheela Crasta, Saroja Kolluru, Maurizio Morri, Serena Y. Tan, Kyle J. Travaglini, Chenling Xu, Marcela Alcántara-Hernández, Nicole Almanzar, Jane Antony, Benjamin Beyersdorf, Deviana Burhan, Kruti Calcuttawala, Matthew M. Carter, Charles K. F. Chan, Charles A. Chang, Stephen Chang, Alex Colville, Rebecca N. Culver, Ivana Cvijović, Gaetano D’Amato, Camille Ezran, Francisco X. Galdos, Astrid Gillich, William R. Goodyer, Yan Hang, Alyssa Hayashi, Sahar Houshdaran, Xianxi Huang, Juan C. Irwin, SoRi Jang, Julia Vallve Juanico, Aaron M. Kershner, Soochi Kim, Bernhard Kiss, William Kong, Maya E. Kumar, Angera H. Kuo, Rebecca Leylek, Baoxiang Li, Gabriel B. Loeb, Wan-Jin Lu, Sruthi Mantri, Maxim Markovic, Patrick L. McAlpine, Antoine de Morree, Karim Mrouj, Shravani Mukherjee, Tyler Muser, Patrick Neuhöfer, Thi D. Nguyen, Kimberly Perez, Ragini Phansalkar, Nazan Puluca, Zhen Qi, Poorvi Rao, Hayley Raquer-McKay, Nicholas Schaum, Bronwyn Scott, Bobak Seddighzadeh, Joe Segal, Sushmita Sen, Shaheen Sikandar, Sean P. Spencer, Lea Steffes, Varun R. Subramaniam, Aditi Swarup, Michael Swift, Will Van Treuren, Emily Trimm, Stefan Veizades, Sivakamasundari Vijayakumar, Kim Chi Vo, Sevahn K. Vorperian, Wanxin Wang, Hannah N. W. Weinstein, Juliane Winkler, Timothy T. H. Wu, Jamie Xie, Andrea R. Yung, Yue Zhang, Angela M. Detweiler, Honey Mekonen, Norma F. Neff, Rene V. Sit, Michelle Tan, Jia Yan, Gregory R. Bean, Vivek Charu, Erna Forgó, Brock A. Martin, Michael G. Ozawa, Oscar Silva, Angus Toland, Venkata N. P. Vemuri, Shaked Afik, Kyle Awayan, Rob Bierman, Olga Borisovna Botvinnik, Ashley Byrne, Michelle Chen, Roozbeh Dehghannasiri, Adam Gayoso, Alejandro A. Granados, Qiqing Li, Gita Mahmoudabadi, Aaron McGeever, Julia Eve Olivieri, Madeline Park, Neha Ravikumar, Geoff Stanley, Weilun Tan, Alexander J. Tarashansky, Rohan Vanheusden, Peter Wang, Sheng Wang, Galen Xing, Chenling Xu, Nir Yosef, Rebecca Culver, Les Dethlefsen, Po-Yi Ho, Shixuan Liu, Jonathan S. Maltzman, Ross J. Metzger, Koki Sasagawa, Rahul Sinha, Hanbing Song, Bruce Wang, Steven E. Artandi, Philip A. Beachy, Michael F. Clarke, Linda C. Giudice, Franklin W. Huang, Kerwyn Casey Huang, Juliana Idoyaga, Seung K. Kim, Christin S. Kuo, Patricia Nguyen, Thomas A. Rando, Kristy Red-Horse, Jeremy Reiter, David A. Relman, Justin L. Sonnenburg, Albert Wu, Sean M. Wu, Tony Wyss-Coray, Stephen R. Quake

**Affiliations:** 1 Department of Chemical Engineering, Stanford University, Stanford, CA USA; 2 ChEM-H, Stanford University, Stanford, CA USA; 3 Department of Bioengineering, Stanford University, Stanford, CA USA; 4 Department of Applied Physics, Stanford University, Stanford, CA USA; 5 Chan Zuckerberg Biohub, San Francisco, CA USA; 6 Chan Zuckerberg Biohub, San Francisco, CA USA; 7 Department of Biochemistry, Stanford University School of Medicine, Stanford, CA USA; 8 Howard Hughes Medical Institute, San Francisco CA, USA; 9 Department of Biomedical Data Science, Stanford University, Stanford, CA USA; 10 Center for Computational Biology, University of California, Berkeley, Berkeley, CA USA; 11 Department of Electrical Engineering and Computer Sciences, University of California, Berkeley, Berkeley, CA USA; 12 Ragon Institute of MGH, MIT and Harvard, Cambridge, MA USA; 13 Donor Network West, San Ramon, CA USA; 14 Department of Otolaryngology-Head and Neck Surgery, Stanford University School of Medicine, Stanford, CA USA; 15 Department of Surgery, University of California, San Francisco, San Francisco, CA USA; 16 Diabetes Center, University of California, San Francisco, San Francisco, CA USA; 17 DCI Donor Services, Sacramento, CA USA; 18 Department of Pathology, Stanford University School of Medicine, Stanford, CA USA; 19 Department of Microbiology and Immunology, Stanford University School of Medicine, Stanford, CA USA; 20 Department of Pediatrics, Division of Pulmonary Medicine, Stanford University, Stanford, CA USA; 21 Institute for Stem Cell Biology and Regenerative Medicine, Stanford University School of Medicine, Stanford, CA USA; 22 Department of Medicine, Division of Cardiovascular Medicine, Stanford University, Stanford, CA USA; 23 Department of Medicine and Liver Center, University of California, San Francisco, San Francisco, CA USA; 24 Department of Neurology and Neurological Sciences, Stanford University School of Medicine, Stanford, CA USA; 25 Department of Surgery - Plastic and Reconstructive Surgery, Stanford University School of Medicine, Stanford, CA USA; 26 Department of Developmental Biology, Stanford University School of Medicine, Stanford, CA USA; 27 Division of Infectious Diseases & Geographic Medicine, Department of Medicine, Stanford University, School of Medicine, Stanford, CA USA; 28 Department of Genetics, Stanford University School of Medicine, Stanford, CA USA; 29 Department of Biology, Stanford University, Stanford, CA USA; 30 Department of Pediatrics, Division of Cardiology, Stanford University School of Medicine, Stanford, CA USA; 31 Stanford Diabetes Research Center, Stanford University School of Medicine, Stanford, CA USA; 32 Center for Gynecology and Reproductive Sciences, Department of Obstetrics, Gynecology and, Reproductive Sciences, University of California, San Francisco, San Francisco, CA USA; 33 Department of Critical Care Medicine, The First Affiliated Hospital of Shantou University Medical College, Shantou, China; 34 Department of Ophthalmology, Stanford University School of Medicine, Stanford, CA USA; 35 Division of Nephrology, Department of Medicine, University of California, San Francisco, San Francisco, CA USA; 36 Stanford University School of Medicine, Stanford, CA USA; 37 Mass Spectrometry Platform, Chan Zuckerberg Biohub, Stanford, CA USA; 38 Stanford Cancer Institute, Stanford University School of Medicine, Stanford, CA USA; 39 Department of Medicine, Division of Hematology, Stanford University School of Medicine, Stanford, CA USA; 40 Department of Biochemistry and Biophysics, Cardiovascular Research Institute, University of California, San Francisco, San Francisco, CA USA; 41 Division of Hematology and Oncology, Department of Medicine, Bakar Computational Health Sciences Institute, Institute for Human Genetics, University of California, San Francisco, San Francisco, CA USA; 42 Stanford Cardiovascular Institute, Stanford, CA USA; 43 Department of Chemical and Systems Biology, Stanford University School of Medicine, Stanford, CA USA; 44 Department of Cell & Tissue Biology, University of California, San Francisco, San Francisco, CA USA; 45 Institute for Computational and Mathematical Engineering, Stanford University, Stanford, CA USA; 46 Paul F. Glenn Center for the Biology of Aging, Stanford University School of Medicine, Stanford, CA USA; 47 Division of Nephrology, Stanford University School of Medicine, Stanford, CA USA; 48 Veterans Affairs Palo Alto Health Care System, Palo Alto, CA USA; 49 Vera Moulton Wall Center for Pulmonary and Vascular Disease, Stanford University School of Medicine, Stanford, CA USA; 50 Department of Urology, Stanford University School of Medicine, Stanford, CA USA; 51 Division of Hematology/Oncology, Department of Medicine, San Francisco Veterans Affairs Health Care System, San Francisco, CA USA; 52 Department of Biochemistry, University of California, San Francisco, San Francisco, CA USA

**Keywords:** Machine learning, Diagnostic markers, Chronic kidney disease, Systems analysis, Next-generation sequencing

## Abstract

Cell-free RNA from liquid biopsies can be analyzed to determine disease tissue of origin. We extend this concept to identify cell types of origin using the Tabula Sapiens transcriptomic cell atlas as well as individual tissue transcriptomic cell atlases in combination with the Human Protein Atlas RNA consensus dataset. We define cell type signature scores, which allow the inference of cell types that contribute to cell-free RNA for a variety of diseases.

## Abstract (web summary)

Cell types affected by various diseases are inferred from cell-free RNA.

## Main

Cell-free RNA (cfRNA) represents a mixture of transcripts reflecting the health status of multiple tissues^1^, thereby affording broad clinical utility. Existing applications span oncology and bone marrow transplantation^2,3^, obstetrics^1,4,5^, neurodegeneration^6^ and liver disease^7^. However, several aspects about the physiologic origins of cfRNA, including the contributing cell types of origin, remain unknown, and current assays focus on tissue-level contributions at best^1,3,4,5–7^. Incorporating knowledge from cellular pathophysiology, which often forms the basis of disease^8^, into a liquid biopsy would more closely match the resolution afforded by invasive procedures.

**Fig. 1:**
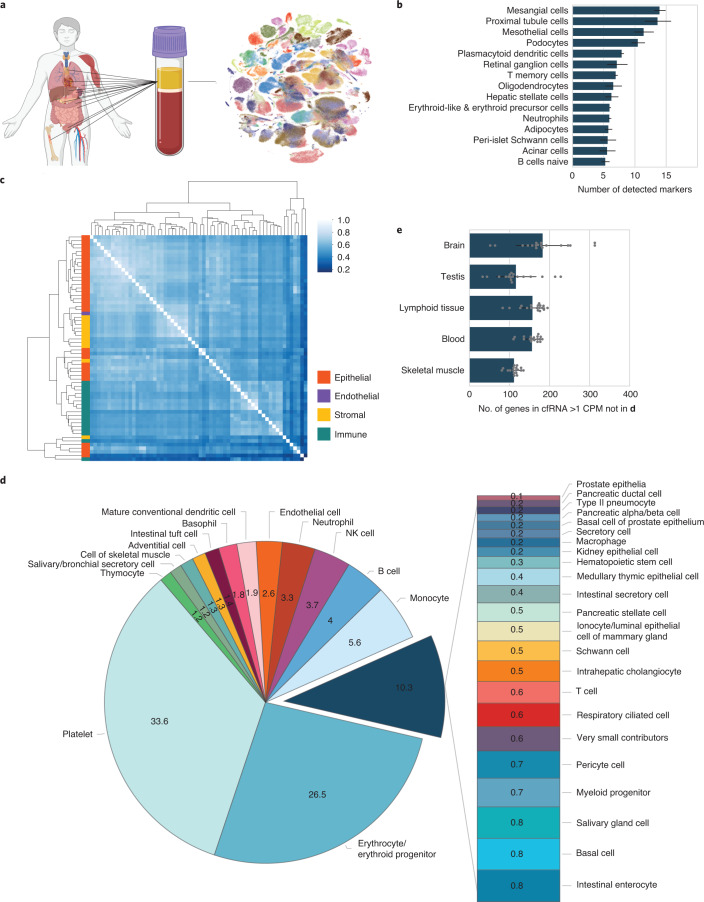
Cell type decomposition of the plasma cell-free transcriptome using Tabula Sapiens. **a**, Integration of tissue of origin and single-cell transcriptomics to identify cell types of origin in cfRNA. **b**, Cell-type-specific markers defined in context of the human body identified in plasma cfRNA. Error bars denote the s.d. of number of cell-type-specific markers (*n* = 75 patients); the measure of center is the mean. CPM-TMM counts for a given gene across technical replicates were averaged before intersection. **c**, Cluster heat map of Spearman correlations of the cell type basis matrix column space derived from Tabula Sapiens. Color bar denotes correlation value. **d**, Mean fractional contributions of cell-type-specific RNA in the plasma cell-free transcriptome (*n* = 18 patients). **e**, Top tissues in cfRNA not captured by basis matrix (the set difference of all genes detected in a given cfRNA sample and the row space of the basis matrix intersection with HPA tissue-specific genes). Error bars denote the s.d. of number of HPA tissue-specific genes with NX counts >10 and cell-free CPM expression ≥ 1 (*n* = 18 patients); the measure of center is the mean.

**Extended Data Fig. 1:**
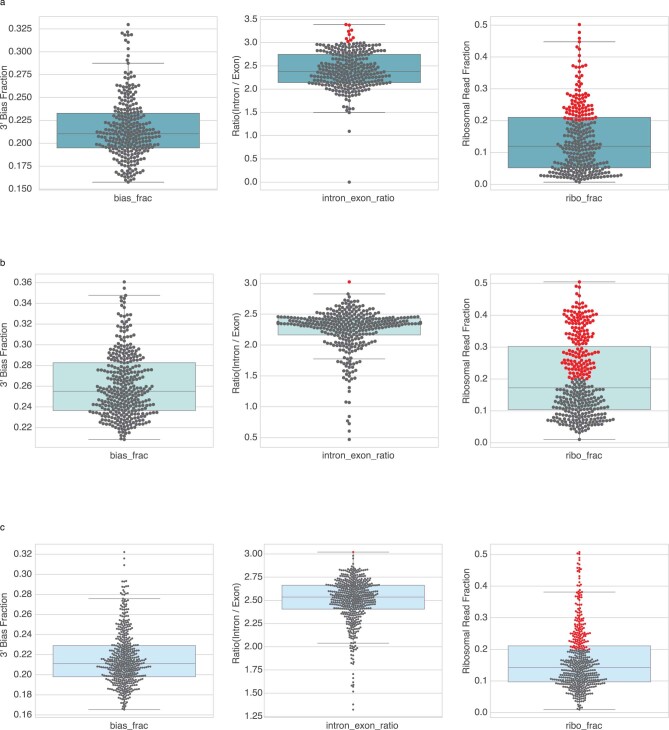
Cell-free RNA Sample Quality Control. Quality control metrics (3′ bias fraction, ribosomal fraction, and DNA contamination) were determined for each cfRNA sample downloaded from a given SRA accession number. Samples with outlier values are highlighted in red and were not considered in subsequent analyses (see Methods section ‘Sample quality filtering’). **(a)** Ibarra et al (n = 285) **(b)** Toden et al (n = 339) **(c)** Chalasani et al (n = 500). Box plot: horizonal line, median; lower hinge, 25^th^ percentile; upper hinge, 75^th^ percentile; whiskers span the 1.5 interquartile range; points outside the whiskers indicate outliers. Each point corresponds to a downloaded cfRNA sample from the corresponding SRA accession number.

We first characterized the landscape of cell-type-specific signal from healthy donor plasma using published exome-enriched cell-free transcriptome data^6^ (Fig. 1a). After removing low-quality samples (Extended Data Fig. 1 and Methods), we intersected the set of genes detected in healthy individuals (*n* = 75) with a database of cell-type-specific markers defined in context of the whole body^9^. Marker genes for blood, brain, and liver cell types were readily detected, as previously observed at tissue level^1,3,4,6,7^, as well as the kidney, gastrointestinal tract, and pancreas (Fig. 1b).

**Extended Data Fig. 2:**
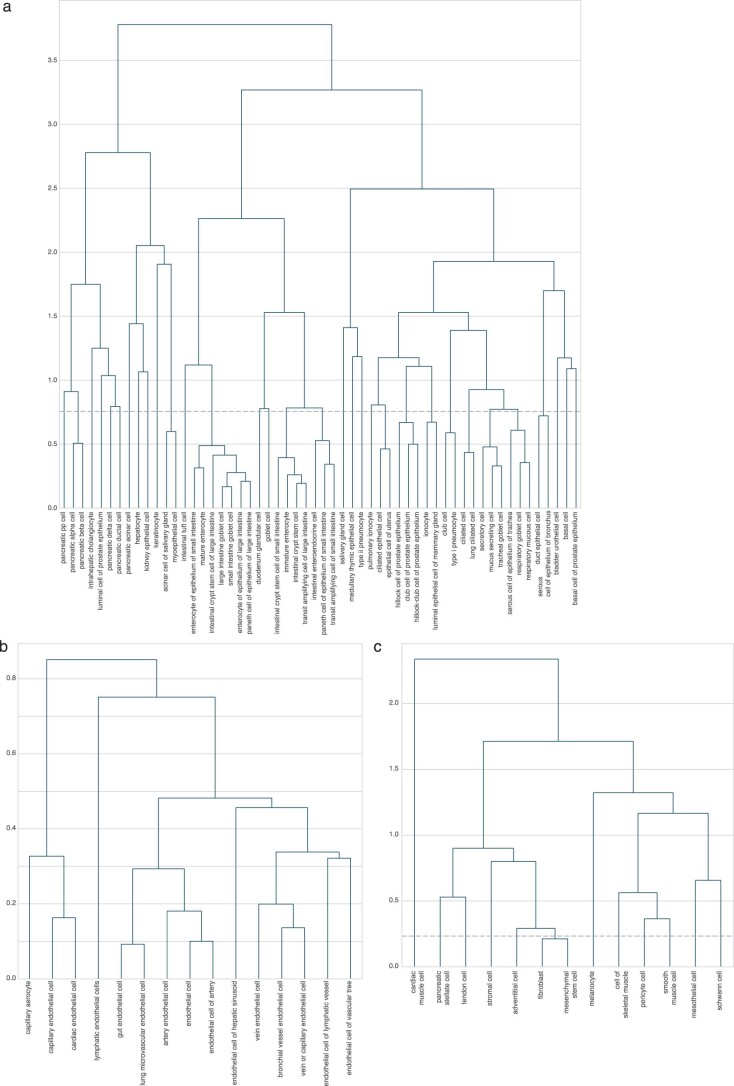
Hierarchical clustering on non-immune Tabula Sapiens organ compartments. Dashed line indicates the height at which tree was cut. Dendrograms correspond with the cell type annotations belonging to **(a)** the epithelial compartment, **(b)** the endothelial compartment **(c)** the stromal compartment.

**Extended Data Fig. 3:**
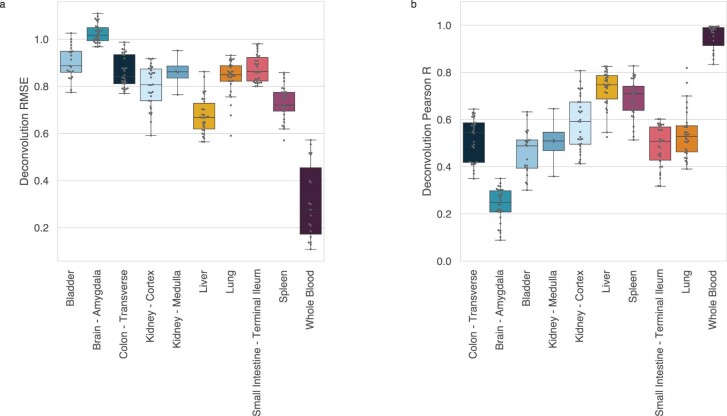
Tabula Sapiens basis matrix performance on GTEx bulk RNA samples using nu-SVR. GTEx tissue samples possessing cell types wholly present and absent from the basis matrix column space were selected. For box plots: horizonal line, median; lower hinge, 25^th^ percentile; upper hinge, 75^th^ percentile; whiskers, 1.5 interquartile range; points outside the whiskers indicate outliers. There are 30 bulk RNA seq samples for a given tissue except for the Bladder (n = 21), Kidney – Medulla (n = 4), and Whole Blood (n = 19). **(a)** Root mean square error between predicted expression and measured expression in a given GTEx tissue. Units are zero-mean unit variance scaled CPM counts. Tissues present in TSP have reduced RMSE compared to those that are absent (Kidney – Medulla and Brain). Tissues with high cellular heterogeneity (for example Lung, Bladder, Small Intestine, Kidney) exhibit reduced deconvolution performance compared to less heterogeneous tissues (for example Whole Blood, Spleen, Liver). **(b)** Pearson correlation between predicted expression and measured expression in a given GTEx tissue.

We then sought to deconvolve the fractions of cell-type-specific RNA using support vector regression, a deconvolution method previously applied to decompose bulk tissue transcriptomes into fractional cell type contributions^10,11^. We used Tabula Sapiens version 1.0 (TSP)^12^, a multiple-donor whole-body cell atlas spanning 24 tissues and organs, to define a basis matrix whose gene set accurately and simultaneously resolved the distinct cell types in TSP. The basis matrix was defined using the gene space that maximized linear independence of the cell types and does not include the whole transcriptome but rather the minimum discriminatory gene set to distinguish between the cell types in TSP. To reduce multicollinearity, transcriptionally similar cell types were grouped (Extended Data Fig. 2). We observed that the basis matrix defined by this gene set appropriately described cell types as most similar to others from the same organ compartment and corresponded to the highest off-diagonal similarity (Fig. 1c). We also confirmed that the basis matrix accurately deconvolved cell-type-specific RNA fractional contributions from several bulk tissue samples^13^ (Extended Data Fig. 3 and Supplementary Information).

**Extended Data Fig. 4:**
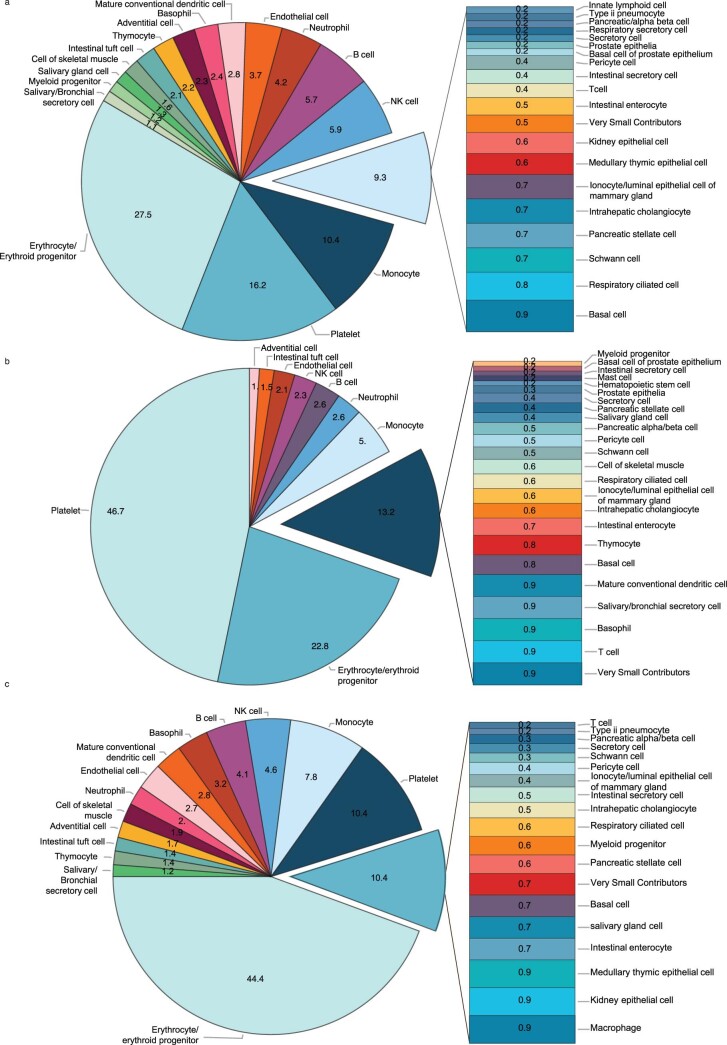
Deconvolution of healthy plasma samples from Toden et al using Tabula Sapiens. Pie charts denote mean fractional cell type specific RNA contributions for **(a)** University of Indiana (n = 17), **(b)** University of Kentucky (n = 18), **(c)** Washington University in St. Louis (n = 22).

**Extended Data Fig. 5:**
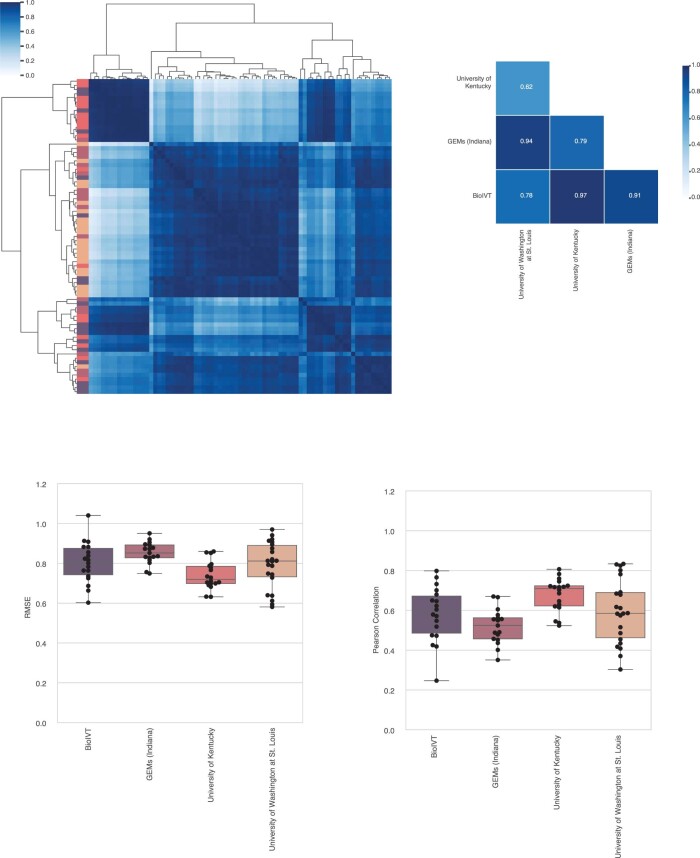
nuSVR decomposition of the plasma cell free transcriptome with Tabula Sapiens. For boxplots, horizonal line, median; lower hinge, 25^th^ percentile; upper hinge, 75^th^ percentile; whiskers span the 1.5 interquartile range; points outside the whiskers indicate outliers. Each point corresponds to a patient in a given cohort; University of Indiana (n = 17), University of Kentucky (n = 18), Washington University in St. Louis (n = 22), and BioIVT (n = 18). For heatmaps or clustermaps, the scale bar denotes the pearson correlation value. **(a)** Complete linkage clustermap of pairwise pearson correlation of deconvolved cell type fractions between patients from a given center; row color denotes a given center (n = 75 patients). **(b)** Heatmap of pairwise pearson correlation of the mean cell type coefficients per center. **(c)** Deconvolution RMSE between predicted vs. measured expression for all biological replicates across all centers. **(d)** Deconvolution pearson correlation between predicted vs. measured expression for all biological replicates across all centers.

We used this matrix to deconvolve the cell types of origin in the plasma cell-free transcriptome (Fig. 1d and Extended Data Figs. 4 and 5). Platelets, erythrocyte/erythroid progenitors and leukocytes comprised the majority of observed signal, whose respective proportions were generally consistent with recent estimates from serum cfRNA^2^ and plasma cfDNA^14^. Within this set of cell types, we suspect that the observation of platelets as a majority cell type, rather than megakaryocytes^2^, likely reflects annotation differences in reference data. We observed distinct transcriptional contributions from solid tissue-specific cell types from the intestine, liver, lungs, pancreas, heart, and kidney (Fig. 1d and Extended Data Fig. 4). Altogether, the observation of contributions from many non-hematopoietic cell types underscores the ability to simultaneously non-invasively resolve contributions to cfRNA from disparate cell types across the body.

Some cell types likely present in the plasma cell-free transcriptome were missing in this decomposition because the source tissues were not represented in TSP. Although, ideally, reference gene profiles for all cell types would be simultaneously considered in this decomposition, a complete reference dataset spanning the entire cell type space of the human body does not yet exist. To identify cell type contributions possibly absent from this analysis, we intersected the genes measured in cfRNA missing from the basis matrix with tissue-specific genes from the Human Protein Atlas (HPA) RNA consensus dataset^15^. This identified both the brain and the testis as tissues whose cell types were not found during systems-level deconvolution and additional genes specific to the blood, skeletal muscle and lymphoid tissues that were not used by the basis matrix (Fig. 1e and Methods).

**Fig. 2:**
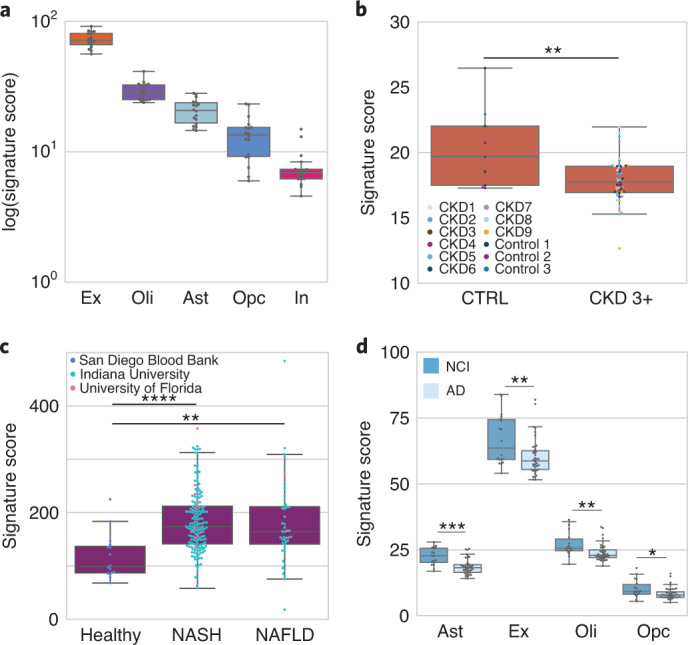
Cellular pathophysiology is non-invasively resolvable in cfRNA. For a given box plot, any cell type signature score is the sum of log-transformed CPM-TMM normalized counts. The horizontal line denotes the median; the lower hinge indicates the 25th percentile; the upper hinge indicates the 75th percentile; whiskers indicate the 1.5 interquartile range; and points outside the whiskers indicate outliers. All *P* values were determined by a Mann–Whitney *U*-test; sidedness is specified in the subplot caption. *P < 0.05, **P < 10^−2^, ***P < 10^−4^, ****P < 10^−5^. **a**, Neuronal and glial cell type signature scores in healthy cfRNA plasma (*n* = 18) on a logarithmic scale. **b**, Comparison of the proximal tubule signature score in CKD stages 3+ (*n* = 51 samples; nine patients) and healthy controls (*n* = 9 samples; three patients) (*P* = 9.66 × 10^−3^, *U* = 116, one sided). Dot color denotes each patient. **c**, Hepatocyte signature score between healthy (*n* = 16) and both NAFLD (*n* = 46) (*P* = 3.15 × 10^−4^, *U* = 155, one sided) and NASH (*n* = 163) (*P* = 4.68 × 10^−6^, *U* = 427, one sided); NASH versus NAFLD (*P* = 0.464, *U* = 3483, two sided). Color reflects sample collection center. **d**, Neuronal and glial signature scores in AD (*n* = 40) and NCI (*n* = 18) cohorts. Excitatory neuron (*P* = 4.94 × 10^−3^, *U* = 206, one sided), oligodendrocyte (*P* = 2.28 × 10^−3^, *U* = 178, two sided), oligodendrocyte progenitor (*P* = 2.27 × 10^−2^, *U* = 224, two sided) and astrocyte (*P* = 6.11 × 10^−5^, *U* = 121, two sided). Ast, astrocyte; Ex, excitatory neuron; In, inhibitory neuron; Oli, oligodendrocyte; Opc, oligodendrocyte precursor cell. Source data

**Extended Data Fig. 6:**
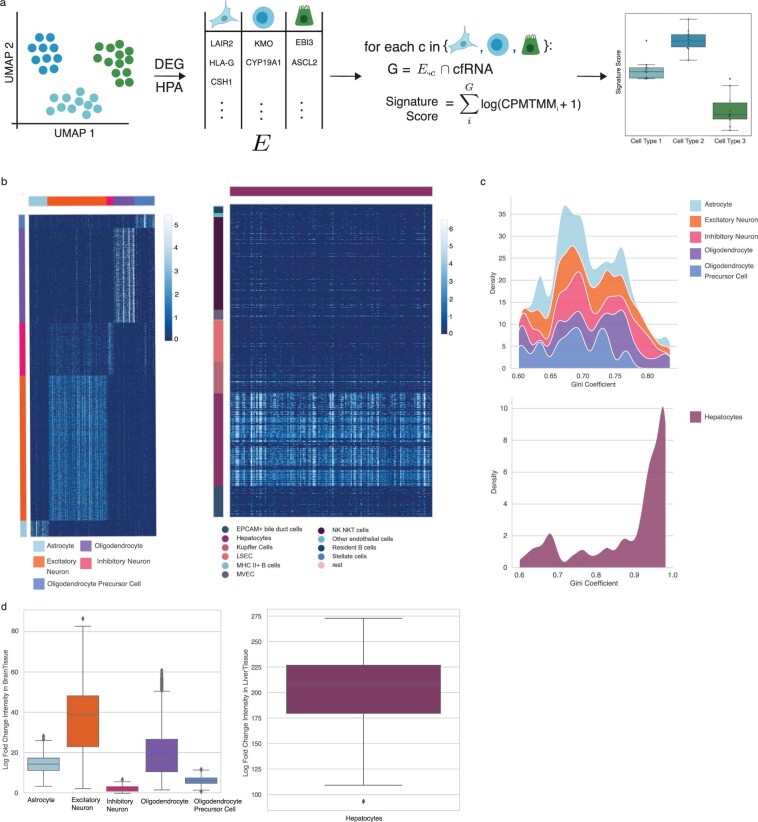
Establishing gene profile cell type specificity in context of the whole body using single cell and bulk RNA-seq data. **(a)** Cell type signature scoring procedure; please see the ‘Signature Scoring’ in the Methods for the full derivation procedure of a given cell type gene profile. **(b)** Single cell heatmaps for gene cell type profiles within the corresponding tissue cell atlas, demonstrating that a cell type specific profile is unique to a given cell type across those within a given tissue. Columns denote marker genes for a given cell type; rows indicate individual cells. The color bar scale corresponds to log-transformed counts-per-ten thousand. **(c)** Gini coefficient density plot for genes in cell type profiles derived from brain and liver single cell atlases using HPA NX counts. The area under the curve for a given cell type sums to one. **(d)** Log fold change in bulk RNA-seq data of a given cell type profile, demonstrating that the predominant expression of the cell type signature in its native tissue is highest relative to other non-native tissues. Values are the log-fold change of the signature score of a given cell type profile in the native tissue (indicated by the y-axis) to the mean expression in the remaining non-native tissues. Box plot: horizontal line, median; lower hinge, 25th percentile; upper hinge, 75th percentile; whiskers span the 1.5 interquartile range; points outside the whiskers indicate outliers (n = 2462 GTEx brain samples for box plot on left; n = 226 GTEx liver samples, right).

**Extended Data Fig. 7:**
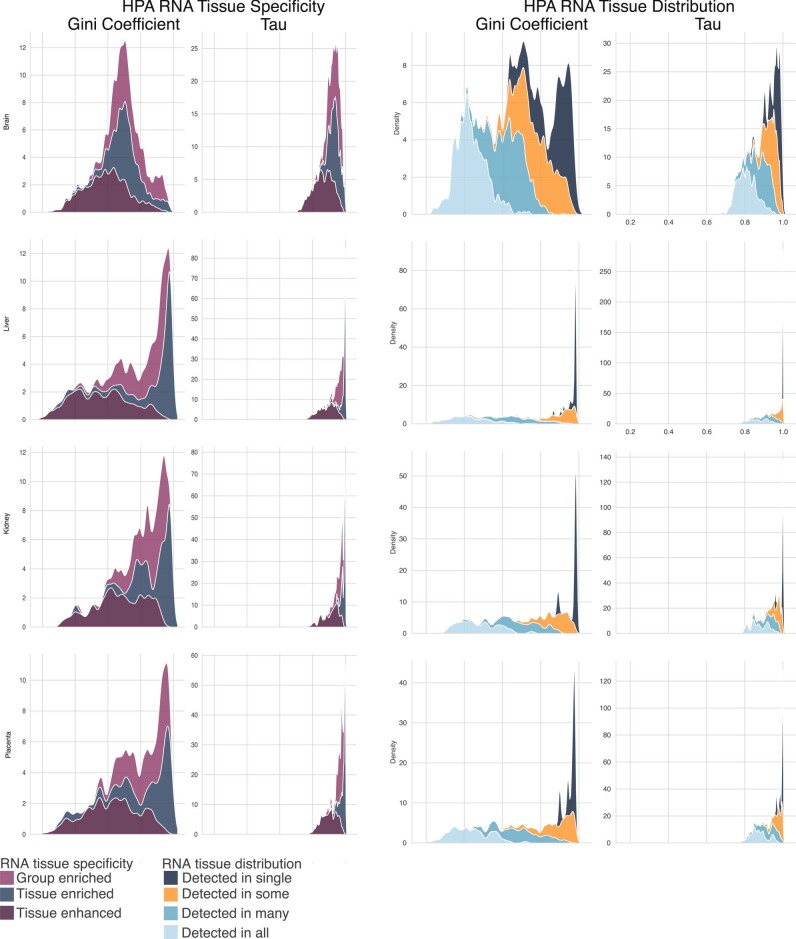
Distribution of Gini coefficient and Tau for all genes denoted by HPA as specific to the brain, liver, placenta, and kidney.

**Extended Data Fig. 8:**
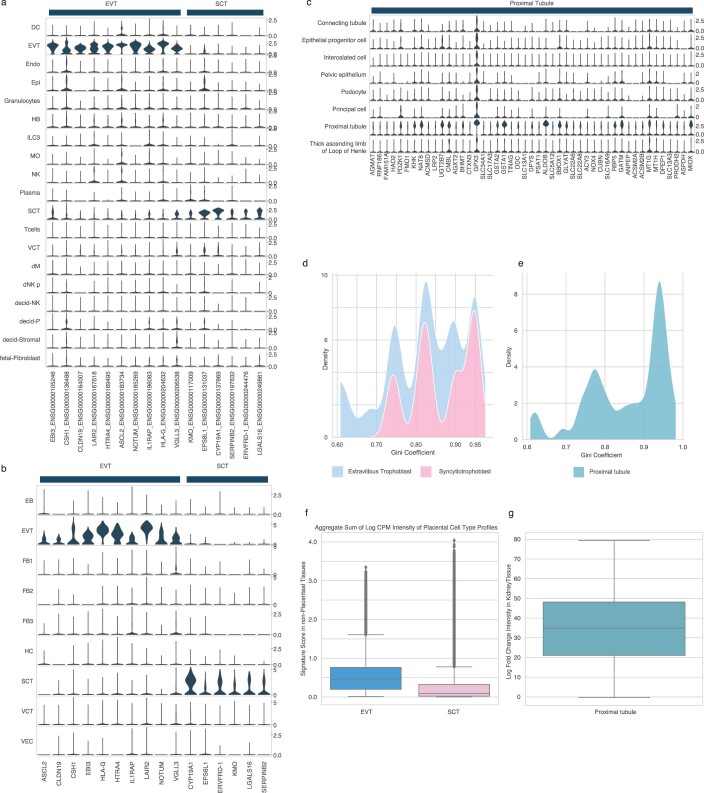
Comprehensive placental and renal cell type gene profile specificity at single cell and whole body resolution. For box plots in f, g: horizontal line, median; lower hinge, 25th percentile; upper hinge, 75th percentile; whiskers span the 1.5 interquartile range; points outside whiskers indicate outliers. **(a)** Violin plot of derived syncytiotrophoblast and extravillous trophoblast gene profiles from Vento-Tormo et al. **(b)** Violin plot of derived syncytiotrophoblast and extravillous trophoblast gene profiles from Suryawanshi et al. **(c)** Violin plot of derived proximal tubule gene profile **(d)** Gini coefficient distribution for placental trophoblast cell types in **(a)** and **(b) (e)** Gini coefficient distribution for renal cell type in **(c) (f)** Distribution of placental trophoblast signature scores across all GTEx tissues. Note: given that the placenta is not in GTEx, the box plots correspond to the distribution of signature scores across non-placental tissues (sum of log-transformed counts-per-ten thousand) (n = 17382 non-placenta GTEx samples) **(g)** Log-fold change of renal cell type signature score in GTEx Kidney Cortex/Medulla samples relative to the mean non-kidney signature score, demonstrating that the predominant expression of the cell type signature in its native tissue is highest relative to other non-native tissues. Values are the log ratio of the signature score in the kidney to the mean signature score in the remaining non-kidney GTEx tissue samples (n = 89 GTEx renal cortex or medulla samples).

As an example of how to analyze cell type contributions from tissues that were not present in TSP, we used an independent brain single-cell atlas along with HPA to define cell type gene profiles and examined their expression in cfRNA (Fig. 2a and Extended Data Figs. 6 and 7). There was a strong signature score from excitatory neurons and a reduced signature score from inhibitory neurons. We observed strong signals from astrocytes, oligodendrocytes and oligodendrocyte precursor cells. These glial cells facilitate brain homeostasis, form myelin and provide neuronal structure and support^8^, consistent with evidence of RNA transport across and the permeability of the blood–brain barrier^16,17^ and that some brain regions are in direct contact with the blood^18^. Similarly, we used published cell atlases for the placenta^19,20^, kidney^21^ and liver^22^ to define cell-type-specific gene profiles (Extended Data Figs. 6 and 8) for signature scoring. These observations augment the resolution of previously observed tissue-specific genes reported to date in cfRNA^1–7^ and formed a baseline from which to measure aberrations in disease.

**Extended Data Fig. 9:**
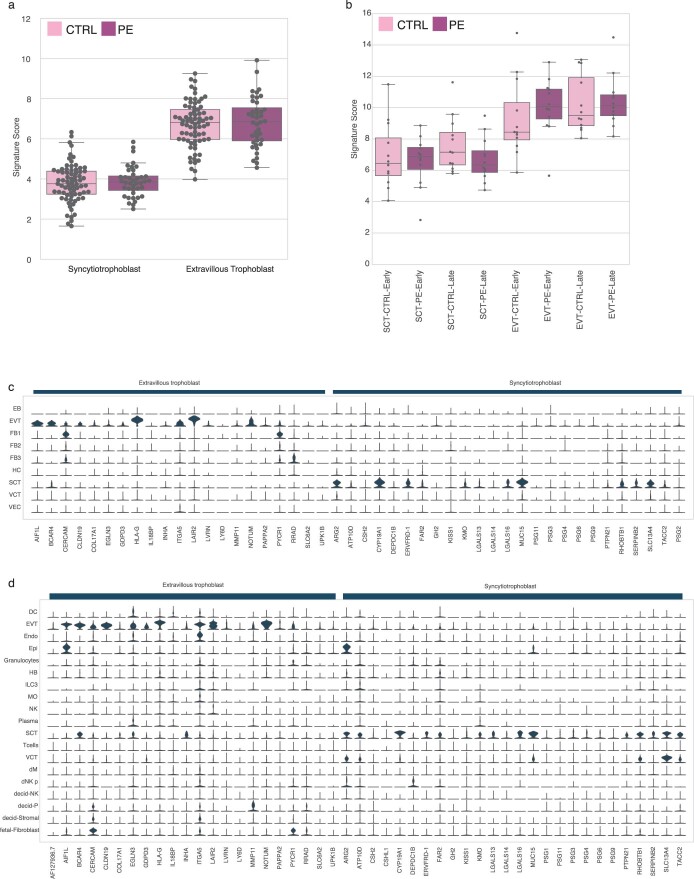
Expression distribution of Tsang et al trophoblast gene profiles in placenta scRNA atlases and in preeclampsia cfRNA. Derived trophoblast signature scores in the **(a)** iPEC dataset (mothers with no complications, n = 73 patients; mothers with preeclampsia, n = 40 patients) and **(b)** PEARL-PEC (n = 12 patients for each early/late-onset PE cohorts and gestationally- age matched healthy controls) datasets from Munchel et al. Box plot: horizontal line, median; lower hinge, 25^th^ percentile; upper hinge, 75^th^ percentile; whiskers span the 1.5 interquartile range; points outside the whiskers indicate outliers. Stacked violin plot of the genes comprising the extravillous trophoblast and syncytiotrophoblast gene profiles from Tsang et al. intersecting with the measured genes in **(c)** Suryawanshi et al and **(d)** Vento-Tormo et al, reflecting the expression distribution across all observed placental cell types.

Cell-type-specific changes drive disease etiology^8^, and we asked whether cfRNA reflected cellular pathophysiology. We considered trophoblasts in preeclampsia^23,24^, proximal tubules in chronic kidney disease (CKD)^25,26^, hepatocytes in non-alcoholic steatohepatitis (NASH)/non-alcoholic fatty liver disease (NAFLD)^27^ and multiple brain cell types in Alzheimer’s disease (AD)^28,29^. As an example of why whole-body cell type characterization is relevant, we observed that a previous attempt to infer trophoblast cell types from cfRNA in preeclampsia^24^ used genes that are not specific or readily measurable within their asserted cell type (Extended Data Fig. 9 and Supplementary Information). However, we found several other cases where cellular pathophysiology can be measured in cfRNA.

**Extended Data Fig. 10:**
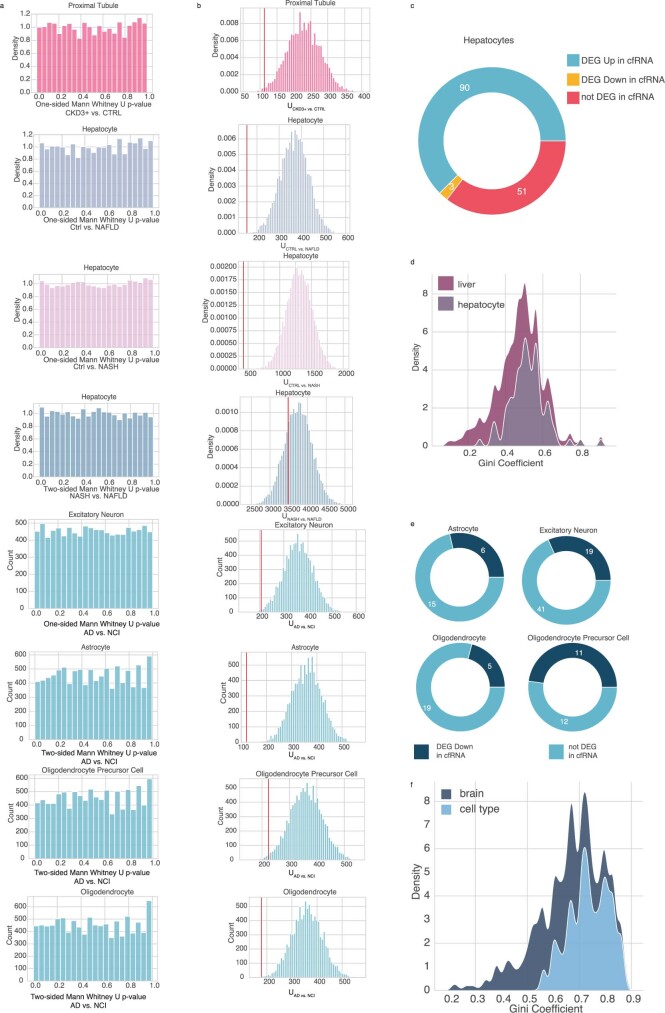
Assessment of cell type gene profile discriminatory power during signature scoring. **(a)** Density of p-values over 10,000 trial permutation test to assess p-value calibration for a given signature score. In all cases, the distribution is uniform, as expected under the null. **(b)** Density of U values over 10,000 trial permutation test; red line indicates the U value corresponding to the experimental comparison reported in Fig. 2. **(c)** Donut plot reflecting the number of genes in the hepatocyte cell type gene profile that intersect with the reported NAFLD DEG in Chalasani et al. **(d)** Density plot reflecting the Gini coefficient distribution corresponding to DEG in NAFLD that are liver or hepatocyte specific. The Gini coefficient is computed using the mean expression per liver cell type in Aizarani et al (Methods). Area under each curve sums to one. **(e)** Donut plots reflecting the number of genes in brain cell type gene profiles that intersect with the reported AD DEG in Toden et al. **(f)** Density plot reflecting the Gini coefficient distribution corresponding to DEG in AD that are brain or brain cell type specific. The Gini coefficient is computed using the mean expression per brain cell type in the ‘Normal’ samples of Mathys et al (Methods). Area under each curve sums to one.

The proximal tubule is a highly metabolic, predominant kidney cell type and is a major source for injury and disease progression in CKD^25,26^. Tubular atrophy is a hallmark of CKD nearly independent of disease etiology^30^ and is superior to clinical gold standard as a predictor of CKD progression^31^. Using data from Ibarra et al., we discovered a striking decrease in the proximal tubule cell signature score of patients with CKD (ages 67–91 years, CKD stage 3–5 or peritoneal dialysis) compared to healthy controls (Fig. 2b and Extended Data Fig. 10a,b). These results demonstrate non-invasive resolution of proximal tubule deterioration observed in CKD histology^31^ and are consistent with findings from invasive biopsy.

Hepatocyte steatosis is a histologic hallmark of NASH and NAFLD phenotypes, whereby the accumulation of cellular stressors results in hepatocyte death^27^. We found that several genes differentially expressed in NAFLD serum cfRNA^7^ were specific to the hepatocyte cell type profile derived above (*P* < 10^−10^, hypergeometric test). Notable hepatocyte-specific differentially expressed genes (DEGs) include genes encoding cytochrome P450 enzymes (including *CYP1A2*, *CYP2E1* and *CYP3A4*), lipid secretion (*MTTP)* and hepatokines (*AHSG* and *LECT2)*^32^. We further observed striking differences in the hepatocyte signature score between healthy and both NAFLD and NASH cohorts and no difference between the NASH and NAFLD cohorts (Fig. 2c and Extended Data Fig. 10).

AD pathogenesis results in neuronal death and synaptic loss^29^. We used brain single-cell data^28^ to define brain cell type gene profiles in both the AD and the normal brain. Several DEGs found in cfRNA analysis of AD plasma are brain cell type specific (*P* < 10^−5^, hypergeometric test). Astrocyte-specific genes include those that encode filament protein (*GFAP*^33^) and ion channels (*GRIN2C*^28^). Excitatory neuron-specific genes encode solute carrier proteins (*SLC17A7*^28^) and *SLC8A2*^34^), cadherin proteins (*CDH8*^35^ and *CDH22*^36^) and a glutamate receptor (*GRM1*^29,37^). Oligodendrocyte-specific genes encode proteins for myelin sheath stabilization (*MOBP*^29^) and a synaptic/axonal membrane protein (*CNTN2*^29^). Oligodendrocyte-precursor-cell-specific genes encode transcription factors (*OLIG2*^38^ and *MYT1*^39^), neural growth and differentiation factor (*CSPG5*^40^) and a protein putatively involved in brain extracellular matrix formation (*BCAN*^41^).

We then inferred neuronal death in plasma cfRNA between AD and healthy non-cognitive controls (NCIs) and also observed differences in oligodendrocyte, oligodendrocyte progenitor and astrocyte signature scores (Fig. 2d and Extended Data Fig. 10). The oligodendrocyte and oligodendrocyte progenitor cells signature score directionality agrees with reports of their death and inhibited proliferation in AD, respectively^42^. The observed astrocyte signature score directionality is consistent with the cell type specificity of a subset of reported downregulated DEGs^6^ and reflects that astrocyte-specific changes, which are known in AD pathology^42^, are non-invasively measurable.

Taken together, this work demonstrates consistent non-invasive detection of cell-type-specific changes in human health and disease using cfRNA. Our findings uphold and further augment the scope of previous work identifying immune cell types^2^ and hematopoietic tissues^1,2^ as primary contributors to the cell-free transcriptome cell type landscape. Our approach is complementary to previous work using cell-free nucleosomes^14^, which depends on a more limited set of reference chromatin immunoprecipitation sequencing data, which are largely at the tissue level^43^. Readily measurable cell types include those specific to the brain, lung, intestine, liver, and kidney, whose pathophysiology affords broad prognostic and clinical importance. Consistent detection of cell types responsible for drug metabolism (for example, liver and renal cell types) as well as cell types that are drug targets, such as neurons or oligodendrocytes for Alzheimer’s-protective drugs, could provide strong clinical trial endpoint data when evaluating drug toxicity and efficacy. We anticipate that the ability to non-invasively resolve cell type signatures in plasma cfRNA will both enhance existing clinical knowledge and enable increased resolution in monitoring disease progression and drug response.

## Methods

### Data processing

#### Data acquisition

cfRNA: For samples from Ibarra et al. (PRJNA517339), Toden et al. (PRJNA574438) and Chalasani et al. (PRJNA701722), raw sequencing data were obtained from the Sequence Read Archive with the respective accession numbers. For samples from Munchel et al., processed counts tables were directly downloaded.

For all individual tissue single-cell atlases, Seurat objects or AnnData objects were downloaded or directly received from the authors. Data from Mathys et al. were downloaded with permission from Synapse. The liver Seurat object was requested from Aizarani et al. For the placenta cell atlases, a Seurat object was requested from Suryawanshi et al., and AnnData was requested from Vento-Tormo et al. Kidney AnnData were downloaded (https://www.kidneycellatlas.org, Mature Full dataset).

HPA version 19 transcriptomic data, Genotype-Tissue Expression (GTEx) version 8 raw counts and Tabula Sapiens version 1.0 were downloaded directly.

#### Bioinformatic processing

All analyses were performed using Python (version 3.6.0) and R (version 3.6.1) For each sample for which raw sequencing data were downloaded, we trimmed reads using trimmomatic (version 0.36) and then mapped them to the human reference genome (hg38) with STAR (version 2.7.3a). Duplicate reads were then marked and removed by the MarkDuplicates tool in GATK (version 4.1.1). Finally, mapped reads were quantified using htseq-count (version 0.11.1), and read statistics were estimated using FastQC (version 0.11.8).

The bioinformatic pipeline was managed using snakemake (version 5.8.1). Read and tool performance statistics were aggregated using MultiQC (version 1.7).

#### Sample quality filtering

For every sample for which raw sequencing data were available, we estimated three quality parameters as previously described^44,45^: RNA degradation, ribosomal read fraction and DNA contamination.

RNA degradation was estimated by calculating a 3′ bias ratio. Specifically, we first counted the number of reads per exon and then annotated each exon with its corresponding gene ID and exon number using htseq-count. Using these annotations, we measured the frequency of genes for which all reads mapped exclusively to the 3′-most exon as compared to the total number of genes detected. We approximated RNA degradation for a given sample as the fraction of genes where all reads mapped to the 3′-most exon.

To estimate ribosomal read fraction, we compared the number of reads that mapped to the ribosome (region GL00220.1:105,424–118,780, hg38) relative to the total number of reads (SAMtools view).

To estimate DNA contamination, we used an intron-to-exon ratio and quantified the number of reads that mapped to intronic as compared to exonic regions of the genome.

We applied the following thresholds as previously reported^44^:

- Ribosomal: >0.2
- 3′ Bias Fraction: >0.4
- DNA Contamination: >3

We considered any given sample as low quality if its value for any metric was greater than any of these thresholds, and we excluded the sample from subsequent analysis.

#### Data normalization

All gene counts were adjusted to counts per million (CPM) reads and per milliliter of plasma used. For a given sample, *i* denotes gene index, and *j* denotes sample index:

1 $$\eta _{ij} = \frac{{\mathrm{Gene}_{ij}}}{{({\mathrm{Library}}\,{\mathrm{size}}_j) \times ({\rm{mL}}\,{\mathrm{plasma}}_j)}}\,{{{\mathrm{where}}}}\,{\mathrm{Library}}\,{\mathrm{size}}_j = \mathop {\sum}\limits_i {G_{ij}}$$

For individuals who had samples with multiple technical replicates, these plasma volume CPM counts were averaged before nu support vector regression (nu-SVR) deconvolution.

For all analyses except nu-SVR (all work except Fig. 1d,e), we next applied trimmed mean of M values (TMM) normalization as previously described^46^ using edgeR (version 3.28.1):

2 $$\frac{{\eta _{ij}}}{{TMM_j}}$$

CPM-TMM normalized gene counts across technical replicates for a given biological replicate were averaged for the count tables used in all analyses performed.

Sequencing batches and plasma volumes were obtained from the authors in Toden et al. and Chalasani et al. for per-sample normalization. For samples from Ibarra et al., plasma volume was assumed to be constant at 1 ml, as we were unable to obtain this information from the authors; sequencing batches were confirmed with the authors (personal communication). All samples from Munchel et al. were used to compute TMM scaling factors, and 4.5 ml of plasma^5^ was used to normalize all samples within a given dataset (both PEARL-PEC and iPEC).

### Cell type marker identification using PanglaoDB

The PanglaoDB cell type marker database was downloaded on 27 March 2020. Markers were filtered for human (‘Hs’) only and for PanglaoDB’s defined specificity (how often marker was not expressed in a given cell type) and sensitivity (how frequently marker is expressed in cells of this type). Gene synonyms from Panglao were determined using MyGene version 3.1.0 to ensure full gene space.

We then intersected this gene space with a cohort of healthy cfRNA samples (*n* = 75, NCI individuals from Toden et al.). A given cell type marker was counted in a given healthy cfRNA sample if its gene expression was greater than zero in log +1 transformed CPM-TMM gene count space.

Cell types with markers filtered by sensitivity = 0.9 and specificity = 0.2 and samples with >5 cell type markers on average are shown in Fig. 1b.

### Basis matrix formation

Scanpy^47^ (version 1.6.0) was used. Only cells from droplet sequencing (‘10x’) were used in analysis given that a more comprehensive set of unique cell types across the tissues in Tabula Sapiens was available^12^. Disassociation genes as reported^12^ were eliminated from the gene space before subsequent analysis.

Given the non-specificity of the following annotations (for example, other cell type annotations at finer resolution existed), cells with these annotations were excluded from subsequent analysis:

- ‘epithelial cell’
- ‘ocular surface cell’
- ‘radial glial cell’
- ‘lacrimal gland functional unit cell’
- ‘connective tissue cell’
- ‘corneal keratocyte’
- ‘ciliary body’
- ‘bronchial smooth muscle cell’
- ‘fast muscle cell’
- ‘muscle cell’
- ‘myometrial cell’
- ‘skeletal muscle satellite stem cell’
- ‘slow muscle cell’
- ‘tongue muscle cell’
- ‘vascular associated smooth muscle cell’
- ‘alveolar fibroblast’
- ‘fibroblast of breast’
- ‘fibroblast of cardiac tissue’
- ‘myofibroblast cell’

All additional cells belonging to the ‘Eye’ tissue were excluded from subsequent analysis given discrepancies in compartment and cell type annotations and the unlikelihood of detecting eye-specific cell types. The resulting cell type space still possessed several transcriptionally similar cell types (for example, various intestinal enterocytes, T cells or dendritic cells), which, left unaddressed, would reduce the linear independence of the basis matrix column space and, hence, would affect nu-SVR deconvolution.

Cells were, therefore, assigned broader annotations on a per-compartment basis as follows:

Epithelial, Stromal, Endothelial: Using counts from the ‘decontXcounts’ layer of the adata object, cells were CPM normalized (sc.pp.normalize_total(target_sum = 1 × 10^6^)) and log-transformed (sc.pp.log1p). Hierarchical clustering with complete linkage (sc.tl.dendrogram) was performed per compartment on the feature space comprising the first 50 principal components (sc.pp.pca). Epithelial and stromal compartment dendrograms were then cut (scipy.cluster.hierarchy.cut_tree) at 20% and 10% of the height of the highest node, respectively, such that cell types with high transcriptional similarity were grouped together, but overall granularity of the cell type labels was preserved. This work is available in the script ‘treecutter.ipynb’ on GitHub; the scipy version used is 1.5.1.

The endothelial compartment dendrogram revealed high transcriptional similarity across all cell types (maximum node height = 0.851) compared to epithelial (maximum node height = 3.78) and stromal (maximum node height = 2.34) compartments (Extended Data Fig. 2). To this end, only the ‘endothelial cell’ annotation was used for the ‘endothelial’ compartment.

Immune: Given the high transcriptional similarity and the varying degree of annotation granularity across tissues and cell types, cell types were grouped on the basis of annotation. The following immune annotations were kept:

- ‘b cell’
- ‘basophil’
- ‘erythrocyte’
- ‘erythroid progenitor’
- ‘hematopoietic stem cell’
- ‘innate lymphoid cell’
- ‘macrophage’
- ‘mast cell’
- ‘mature conventional dendritic cell’
- ‘microglial cell’
- ‘monocyte’
- ‘myeloid progenitor’
- ‘neutrophil’
- ‘nk cell’
- ‘plasma cell’
- ‘plasmablast’
- ‘platelet’
- ‘t cell’
- ‘thymocyte’

All other immune compartment cell type annotations were excluded for being too broad when more detailed annotations existed (that is, ‘granulocyte’, ‘leucocyte’ and ‘immune cell’) or present in only one tissue (that is, ‘erythroid lineage cell’; eye, ‘myeloid cell’; and pancreas/prostate). The ‘erythrocyte’ and ‘erythroid progenitor’ annotations were further grouped to minimize multicollinearity.

Using the entire cell type space spanning all four organ compartments, either 30 observations (for example, measured cells) were randomly sampled or the maximum number of available observations (if less than 30) was subsampled, whichever was greater.

Cell type annotations were then reassigned based on the ‘broader’ categories from hierarchical clustering (‘coarsegrain.py’). Raw count values from the DecontX adjusted layer were used to minimize signal spread contamination that could affect DEG analysis^12^.

This subsampled counts matrix was then passed to the ‘Create Signature Matrix’ analysis module at https://cibersortx.stanford.edu/, with the following parameters:

- Disable quantile normalization = True
- Minimum expression = 0.25
- Replicates = 5
- Sampling = 0.5
- Kappa = 999
- *q* value = 0.01
- No. of barcode genes = 3,000–5,000
- Filter non-hematopoietic genes = False

The resulting basis matrix was used in our nu-SVR deconvolution code, available on GitHub, under the name ‘tsp_v1_basisMatrix.txt’.

Abbreviations (left) of grouped cell types (right) in Fig. 1d and the Extended Data are as follows:

- gland cell: ‘acinar cell of salivary gland/myoepithelial cell’
- respiratory ciliated cell: ‘ciliated cell/lung ciliated cell’
- prostate epithelia: ‘club cell of prostate epithelium/hillock cell of prostate epithelium/hillock-club cell of prostate epithelium’
- salivary/bronchial secretory cell: ‘duct epithelial cell/serous cell of epithelium of bronchus’
- intestinal enterocyte: ‘enterocyte of epithelium of large intestine/enterocyte of epithelium of small intestine/intestinal crypt stem cell of large intestine/large intestine goblet cell/mature enterocyte/paneth cell of epithelium of large intestine/small intestine goblet cell’
- intestinal crypt stem cell: ‘immature enterocyte/intestinal crypt stem cell/intestinal crypt stem cell of small intestine/transit amplifying cell of large intestine’
- erythrocyte/erythroid progenitor: ‘erythrocyte/erythroid progenitor’
- fibroblast/mesenchymal stem cell: ‘fibroblast/mesenchymal stem cell’
- intestinal secretory cell: ‘intestinal enteroendocrine cell/paneth cell of epithelium of small intestine/transit amplifying cell of small intestine’
- ionocyte/luminal epithelial cell of mammary gland: ‘ionocyte/luminal epithelial cell of mammary gland’
- secretory cell: ‘mucus secreting cell/secretory cell/tracheal goblet cell’
- pancreatic alpha/beta cell: ‘pancreatic alpha cell/pancreatic beta cell’
- respiratory secretory cell: ‘respiratory goblet cell/respiratory mucous cell/serous cell of epithelium of trachea’
- basal prostate cell: ‘basal cell of prostate epithelia’

### Nu-SVR deconvolution

We formulated the cell-free transcriptome as a linear summation of the cell types from which it originates^1,48^. With this formulation, we adapted existing deconvolution methods developed with the objective of decomposing a bulk tissue sample into its single-cell constituents^10,11^, where the deconvolution problem is formulated as:

3 $$A\theta = b$$

Here, *A* is the representative basis matrix (*g* × *c*) of *g* genes for *c* cell types, which represent the gene expression profiles of the *c* cell types. *θ* is a vector (*c* × 1) of the contributions of each of the cell types, and *b* is the measured expression of the genes observed in blood plasma (*g* × 1). The goal here is to learn *θ* such that the matrix product *Aθ* predicts the measured signal *b*. The derivation of the basis matrix *A* is described in the section ‘Basis matrix formation’.

We performed nu-SVR using a linear kernel to learn *θ* from a subset of genes from the basis matrix to best recapitulate the observed signal *b*, where nu corresponds to a lower bound on the fraction of support vectors and an upper bound on the fraction of margin errors^49^. Here, the support vectors are the genes from the basis matrix used to learn *θ*; *θ* reflects the learned weights of the cell types in the basis matrix column space. For each sample, a set of *θ* was learned by performing a grid search on the two SVR hyperparameters: $\nu \in \{ 0.05,0.1,0.15,0.25,0.5,0.75\}$ and $C \in \{ 0.1,0.5,0.75,1,10\}$.

For each sample, we next enforce two constraints: *θ* can contain only non-negative weights, and the weights in *θ* must sum to 1. Each *θ* corresponding to a hyperparameter combination was normalized as previously described in two steps^10,11^. First, only non-negative weights were kept:

4 $$\forall \theta _j < 0 \in \left\{ {\theta _1, \ldots ,\theta _c} \right\} \to 0$$

Second, the remaining non-zero weights were then normalized by their sum to yield the relative proportions of cell-type-specific RNA.

We then determined the basis matrix dot product with the set of normalized weights for each sample. This dot product yields the predicted expression value for each gene in a given cfRNA mixture with imposed non-negativity on the normalized coefficient vector. The root mean square error (RMSE) was then computed using the predicted expression values and the measured values of these genes for each hyperparameter combination in a given cfRNA mixture. The model yielding the smallest RMSE in predicting expression for a given cfRNA sample was then chosen and assigned as the final deconvolution result for a given sample.

Only CPM counts ≥1 were considered in the mixture, *b*. The values in the basis matrix were also CPM normalized. Before deconvolution, the mixture and basis matrix were centered and scaled to zero mean and unit variance for improved runtime performance. We emphasize that we did not log-transform counts in *b* or in *A*, as this would destroy the requisite linearity assumption in equation (3). Specifically, the concavity of the log function would result in the consistent underestimation of *θ* during deconvolution^50^.

We used the function nu-SVR from scikitlearn^51^ version 0.23.2.

The samples used for nu-SVR deconvolution were 75 NCI patients from Toden et al. spanning four sample collection centers. Given center-specific batch effects reported by Toden et al., we report our results on a per-center basis (Fig. 1d and Extended Data Figs. 4 and 5). There was good pairwise similarity of the learned coefficients among biological replicates within and across sample centers (Extended Data Fig. 5a,b). Deconvolution performance yielded RMSE and Pearson *r* consistent with deconvolved GTEx tissues (Extended Data Fig. 3) whose distinct cell types were in the basis matrix column space (Extended Data Fig. 5c,d). In interpreting the resulting cell type fractions, a limitation of nu-SVR is that it uses highly expressed genes as support vectors and, consequently, assigns a reduced fractional contribution to cell types expressing genes at lower levels or that are smaller in cell volume. Comparison of nu-SVR to quadratic programming^1^ and non-negative linear least squares^52^ yielded similar deconvolution RMSE and Pearson correlation. In contrast to the other methods, nu-SVR cell type contributions were the most consistent with the cell type markers detected using PanglaoDB and was, hence, chosen as the deconvolution model for this work.

### Evaluating basis matrix on GTEx samples

Bulk RNA sequencing samples from GTEx version 8 were deconvolved with the derived basis matrix from tissues that were present (that is, kidney cortex, whole blood, lung and spleen) or absent (for example, kidney medulla and brain) from the basis matrix derived using Tabula Sapiens version 1.0. For each tissue type, the maximum number of available samples or 30 samples, whichever was smaller, was deconvolved. See Supplementary Note 1 for additional discussion.

### Identifying tissue-specific genes in cfRNA absent from basis matrix

To identify cell-type-specific genes in cfRNA that were distinct to a given tissue, we considered the set difference of the non-zero genes measured in a given cfRNA sample with the row space of the basis matrix and intersected this with HPA tissue-specific genes:

5 $$(G_j - R) \cap HPA$$

where *G*_*j*_ is the gene set in the *j*^th^ deconvolved sample, where a given gene in the set’s expression was ≥1 CPM. *R* is the set of genes in the row space of the basis matrix used for nu-SVR deconvolution. *HPA* denotes the total set of tissue-specific genes from HPA.

The HPA tissue-specific gene set (*HPA*) comprised genes across all tissues with Tissue Specificity assignments ‘Group Enriched’, ‘Tissue Enhanced’, ‘Tissue Enriched’ and NX expression ≥10. This approach yielded tissues with several distinct genes present in cfRNA, which could then be subsequently interrogated using single-cell data.

### Derivation of cell-type-specific gene profiles in context of the whole body using single-cell data

For this analysis, only cell types unique to a given tissue (that is, hepatocytes unique to the liver or excitatory neurons unique to the brain) were considered so that bulk transcriptomic data could be used to ensure specificity in context of the whole body. A gene was asserted to be cell type specific if it was (1) differentially expressed within a given single-cell tissue atlas, (2) possessed a Gini coefficient ≥0.6 and was listed as specific to the native tissue for the cell type of interest, indicating comprehensive tissue specificity in context of the whole body (Extended Data Figs. 6 and 8).

1. **Single-cell differential expression**
  For data received as a Seurat object, conversion to AnnData (version 0.7.4) was performed by saving as an intermediate loom object (Seurat version 3.1.5) and converting to AnnData (loompy version 3.0.6). Scanpy (version 1.6.0) was used for all other single-cell analysis. Reads per cell were normalized for library size (scanpy normalize_total, target_sum = 1 × 10^4^) and then logged (scanpy log1p). Differential expression was performed using the Wilcoxon rank-sum test in Scanpy’s filter_rank_genes_groups with the following arguments: min_fold_change = 1.5, min_in_group_fraction = 0.2, max_out_group_fraction = 0.5, corr_method = ‘benjamini-hochberg’. The set of resulting DEGs with Benjamini–Hochberg-adjusted *P* values <0.01 whose ratio of the highest out-group percent expressed to in-group percent expressed <0.5 was selected to ensure high specific expression in the cell type of interest within a given cell type atlas.
2. **Quantifying comprehensive whole-body tissue specificity using the Gini coefficient**
  The distribution of all the Gini coefficiets and Tau values across all genes belonging to cell type gene profiles for cell types native to a given tissue were compared using the HPA gene expression Tissue Specificity and Tissue Distribution assignments^15^ (Extended Data Fig. 7). The Gini coefficient better reflected the underlying distribution of gene expression tissue specificity than Tau (Extended Data Fig. 7) and, hence, were used for subsequent analysis. As the Gini coefficient approaches unity, this indicates extreme gene expression inequality or equivalently high specificity. A single threshold (Gini coefficient ≥ 0.6) was applied across all atlases to facilitate a generalizable framework from which to define tissue-specific cell type gene profiles in context of the whole body in a principled fashion for signature scoring in cfRNA.
  For the following definitions, *n* denotes the total number of tissues, and *x*_*j*_ is the expression of a given gene in the *i*^th^ tissue.
  The Gini coefficient was computed as defined^53^:
  6 $${\mathrm{Gini}} = \frac{{n + 1}}{n} - \frac{{2{\mathop \sum \nolimits_{i = 1}^{n}}\left( {n + 1 - i} \right){x_i}}}{{n{\mathop \sum \nolimits_{i = 1}^{n}}{x_i}}}\, ;\: {x_i}\, {\mathrm{is}}\, {\mathrm{ordered}}\, {\mathrm{from}}\, {\mathrm{least}}\, {\mathrm{to}}\, {\mathrm{greatest}}.$$
  Tau, as defined in ref. ^53^:
  7 $$\tau = \frac{{\mathop {\sum }\nolimits_{i = 1}^n 1 - \bar x}}{{n - 1}}\ {{{\mathrm{where}}}}\,\bar x = \frac{{x_i}}{{{{{\mathrm{max}}}}\left( {x_i} \right)\forall i \in \{ 1 \ldots n\} }}$$
  HPA NX Counts from the HPA object titled ‘rna_tissue_consensus.tsv’ accessed on 1 July 2019 were used for computing Gini coefficients and Tau.
  Note for brain cell type gene profiles: Given that there are multiple sub brain regions in the HPA data, the determined Gini coefficients are lower (for example, not as close to unity compared to other cell type gene profiles) because there are multiple regions of the brain with high expression, which would result in reduced count inequality.

### Gene expression in GTEx

We confirmed the specificity of a given gene profile to its corresponding cell type by comparing the aggregate expression of a given cell type signature in its native tissue compared to that of the average across remaining GTEx tissues (Extended Data Figs. 6d and 8f,g). We uniformly observed a median fold change greater than 1 in the signature score of a cell type gene profile in its native tissue relative to the mean expression in other tissues, confirming high specificity.

Raw GTEx data version 8 (accessed 26 August 2019) were converted to log(counts-per-ten-thousand + 1) counts. The signature score was determined by summing the expression of the genes in a given bulk RNA sample for a given cell type gene profile. Because only gene profiles were derived for cell types that correspond to a given tissue, the mean signature score of a cell type profile across the non-native tissues was then computed and used to determine the log fold change.

### Cell type specificity of DEGs in AD and NAFLD cfRNA

After observing a significant intersection between the DEGs in AD^6^ or NAFLD^7^ in cfRNA with corresponding cell-type-specific genes (Extended Data Fig. 10c,e), we then assessed the cell type specificity of DEGs using a permutation test. To assess whether DEGs that intersected with a cell type gene profile were more specific to a given cell type than DEGs that were generally tissue specific, we performed a permutation test. Specifically, we compared the Gini coefficient for genes in these two groups, computed using the mean expression of a given gene across brain cell types from healthy brain^28^ or liver^22^ single-cell data. We considered the cell type gene profiles as defined for signature scoring in Fig. 2.

The starting set of tissue-specific genes was defined using the HPA tissue transcriptional data annotated as ‘Tissue enriched’, ‘Group enriched’ or ‘Tissue enhanced’ (brain, accessed on 13 January 2021; liver, accessed on 28 November 2020). These requirements ensured the specificity of a given brain/liver gene in context of the whole body. For a given tissue, this formed the initial set of tissue-specific genes *B*.

The union of all brain or liver cell-type-specific genes is the set *C*. All genes in *C* (‘cell type specific’) were a subset of the respective initial set of tissue-specific genes:

8 $$C - B = 0$$

Genes in *B* that did not intersect with *C* and intersected with DEG-up (*U*) or DEG-down (*D*) genes in a given disease^6,7^ were then defined as ‘tissue specific’.

9 $$T = \left( {B \cap U} \right) \cup (B \cap D) - C$$

The Gini coefficients reflecting the gene expression inequality across the cell types within corresponding tissue single-cell atlas were computed for the gene sets labeled as ‘cell type specific’ and ‘tissue specific’. Brain reference data to compute Gini coefficients were from the single-cell brain atlas with diagnosis as ‘Normal’^28^. Liver single cell data were used as-is^22^. All Gini coefficients were computed using the mean log-transformed CPTT (counts per ten thousand) gene expression per cell type.

A permutation test was then performed on the union of the Gini coefficients for the genes labeled as ‘cell type specific’ and ‘tissue specific’. The purpose of this test was to assess probability that the observed mean difference in Gini coefficient for these two groups yielded no difference in specificity (that is, H_0_: $\mu _{{\mathrm{cell}}\,{\mathrm{type}}\,{\mathrm{Gini}}\,{\mathrm{coefficient}}} = \mu _{{\mathrm{tissue}}\,{\mathrm{Gini}}\,{\mathrm{coefficient}}}$).

Gini coefficients were permuted and reassigned to the list of ‘tissue specific’ or ‘cell type specific’ genes, and then the difference in the means of the two groups was computed. This procedure was repeated 10,000 times. The *P* value was determined as follows:

10 $$p = \frac{{\# \,{\mathrm{trials}}\,{\mathrm{with}}\,{\mathrm{permuted}}(\mu _{{\mathrm{cell}}\,{\mathrm{type}}} - \mu _{{\mathrm{tissue}}}) \ge \mu _{{\mathrm{observed}}}}}{{10,000 + 1}}$$

where $$\mu _{\mathrm{observed}}: = (\mu _{{\mathrm{cell}}\,{\mathrm{type}}\,{\mathrm{Gini}}\,{\mathrm{coefficient}}} - \mu _{{\mathrm{tissue}}\,{\mathrm{Gini}}\,{\mathrm{coefficient}}})$$.

The additional 1 in the denominator reflects the original test between the true difference in means (for example, the true comparison yielding *μ*_observed_).

NAFLD: We considered the space of reported NAFLD DEGs in serum^7^. Here, *C* = hepatocyte gene profile, and *B* = the liver-specific genes.

AD: First, we intersected a given cell type gene profile in AD with the equivalent Normal profile for comparative analysis. Genes defined as ‘brain cell type specific’ for signature scoring in Fig. 2d were used in this comparison. Of note, no DEG-up genes intersected with any of the brain cell type signatures in Fig. 2d. Microglia, although often implicated in AD pathogenesis, were excluded given their high overlapping transcriptional profile with non-central-nervous-system macrophages^54^. Inhibitory neurons were also excluded given the low number of cell-type-specific genes intersecting between AD and NCI phenotypes.

### Estimating signature scores for each cell type

The signature score is defined as the sum of the log-transformed CPM-TMM normalized counts per gene asserted to be cell type specific, where *i* denotes the index of the gene in a cell type signature gene profile *G* in the *j*^th^ patient sample:

11 $${\mathrm{Signature}}\,{\mathrm{score}}_j = \mathop {\sum}\limits_i {G_{ij}}$$

#### Preeclampsia

For signature scoring of syncytiotrophoblast and extravillous trophoblast gene profiles in PEARL-PEC and iPEC^5^, a respective cell type gene profile used for signature scoring was derived as described in ‘Derivation of cell-type-specific gene profiles in context of the whole body using single-cell data’ independently using two different placental single-cell datasets^19,20^. Only the intersection of the cell-type-specific gene profiles for a given trophoblast cell type between the two datasets was included in the respective trophoblast gene profile for signature scoring.

#### CKD

We compared the signature score of the proximal tubule in CKD (nine patients; 51 samples) and healthy controls (three patients; nine samples). Given that all patient samples were longitudinally sampled over ~30 d (individual samples were taken on different days), we treated the samples as biological replicates and included all time points because the time scale over which renal cell type changes typically occur is longer than the collection period. The sequencing depth was similar between the CKD and healthy cohorts, although it was reduced in comparison to the other cfRNA datasets used in this work. To account for gene measurement dropout, we required that the expression of a given gene in the proximal tubule gene profile was non-zero in at least one sample in both cohorts. Given that all samples were sequenced together, no batch correction was necessary, facilitating a representative comparison between CKD and healthy cohorts.

#### AD

Microglia, although often implicated in AD pathogenesis, were excluded given their high overlapping transcriptional profile with non-central-nervous-system macrophages^54^. Inhibitory neurons were also excluded given the low number of cell-type-specific genes intersecting between AD and NCI phenotypes. Brain gene profiles as defined in the AD section of ‘Cell type specificity of DEGs in AD and NAFLD cfRNA’ were used.

### Assessing *P* value calibration for a given signature score

Cell type signature scores were tested between control and diseased samples with a Mann–Whitney *U*-test. The resulting *P* values were calibrated with a permutation test. Here, the labels compared in a given test (that is, CKD versus control, AD versus NCI, NAFLD versus control, etc.) were randomly shuffled 10,000 times. We observed a well-calibrated, uniform *P*-value distribution (Extended Data Fig. 10a), validating the experimentally observed test statistics.

### Reporting Summary

Further information on research design is available in the Nature Research Reporting Summary linked to this article

## Online content

Any methods, additional references, Nature Research reporting summaries, source data, extended data, supplementary information, acknowledgements, peer review information; details of author contributions and competing interests; and statements of data and code availability are available at 10.1038/s41587-021-01188-9.

## Supplementary information

Supplementary Information Supplementary Fig. 1 and Supplementary Notes 1 and 2

Reporting Summary

Supplementary Data Values to reproduce box plots in Fig. 9a,b.

## Data Availability

All datasets used for this work are publicly available, were downloaded with permission or were directly requested from the authors. Samples from Ibarra et al. (PRJNA517339), Toden et al. (PRJNA574438) and Chalasani et al. (PRJNA701722) were downloaded from the Sequence Read Archive with the respective accession numbers. Reads were mapped to the reference human genome (hg38). For data from Munchel et al., sample gene count tables were directly downloaded. Tissue gene lists and NX counts were downloaded from the Human Protein Atlas (www.proteinatlas.org, version 19). GTEx raw expression data were directly downloaded (https://www.gtexportal.org/home/datasets, GTEx analysis version 8). Tabula Sapiens was downloaded from the Chan Zuckerberg Biohub (https://tabula-sapiens-portal.ds.czbiohub.org, version 1.0). The brain single-cell data were downloaded with permission from Synapse (https://www.synapse.org/#!Synapse:syn18485175), and associated ROSMAP metadata were downloaded with permission from Synapse (https://www.synapse.org/#!Synapse:syn3157322). The liver Seurat object was requested from Aizarani et al. For the placenta atlases, a Seurat object was requested from Suryawanshi et al., and AnnData were requested from Vento-Tormo et al. Kidney AnnData were downloaded (https://www.kidneycellatlas.org, Mature Full dataset). Source data are provided with this paper.

## Source data

Source Data Fig. 2 Values to reproduce box plots in Fig. 2.

## Code availability

Code for the work in this manuscript is available on GitHub at www.github.com/sevahn/deconvolution.
