# Publisher Correction: Cell types of origin of the cell-free transcriptome

**DOI:** 10.1038/s41587-022-01293-3

**Published:** 2022-03-28

**Authors:** Sevahn K. Vorperian, Mira N. Moufarrej, Tabula Sapiens Consortium, Robert C. Jones, Jim Karkanias, Mark Krasnow, Angela Oliveira Pisco, Stephen R. Quake, Julia Salzman, Nir Yosef, Bryan Bulthaup, Phillip Brown, William Harper, Marisa Hemenez, Ravikumar Ponnusamy, Ahmad Salehi, Bhavani A. Sanagavarapu, Eileen Spallino, Ksenia A. Aaron, Waldo Concepcion, James M. Gardner, Burnett Kelly, Nikole Neidlinger, Zifa Wang, Sheela Crasta, Saroja Kolluru, Maurizio Morri, Serena Y. Tan, Kyle J. Travaglini, Chenling Xu, Marcela Alcántara-Hernández, Nicole Almanzar, Jane Antony, Benjamin Beyersdorf, Deviana Burhan, Kruti Calcuttawala, Matthew M. Carter, Charles K. F. Chan, Charles A. Chang, Stephen Chang, Alex Colville, Rebecca N. Culver, Ivana Cvijović, Gaetano D’Amato, Camille Ezran, Francisco X. Galdos, Astrid Gillich, William R. Goodyer, Yan Hang, Alyssa Hayashi, Sahar Houshdaran, Xianxi Huang, Juan C. Irwin, SoRi Jang, Julia Vallve Juanico, Aaron M. Kershner, Soochi Kim, Bernhard Kiss, William Kong, Maya E. Kumar, Angera H. Kuo, Rebecca Leylek, Baoxiang Li, Gabriel B. Loeb, Wan-Jin Lu, Sruthi Mantri, Maxim Markovic, Patrick L. McAlpine, Antoine de Morree, Karim Mrouj, Shravani Mukherjee, Tyler Muser, Patrick Neuhöfer, Thi D. Nguyen, Kimberly Perez, Ragini Phansalkar, Nazan Puluca, Zhen Qi, Poorvi Rao, Hayley Raquer-McKay, Nicholas Schaum, Bronwyn Scott, Bobak Seddighzadeh, Joe Segal, Sushmita Sen, Shaheen Sikandar, Sean P. Spencer, Lea Steffes, Varun R. Subramaniam, Aditi Swarup, Michael Swift, Will Van Treuren, Emily Trimm, Stefan Veizades, Sivakamasundari Vijayakumar, Kim Chi Vo, Sevahn K. Vorperian, Wanxin Wang, Hannah N. W. Weinstein, Juliane Winkler, Timothy T. H. Wu, Jamie Xie, Andrea R. Yung, Yue Zhang, Angela M. Detweiler, Honey Mekonen, Norma F. Neff, Rene V. Sit, Michelle Tan, Jia Yan, Gregory R. Bean, Vivek Charu, Erna Forgó, Brock A. Martin, Michael G. Ozawa, Oscar Silva, Angus Toland, Venkata N. P. Vemuri, Shaked Afik, Kyle Awayan, Rob Bierman, Olga Borisovna Botvinnik, Ashley Byrne, Michelle Chen, Roozbeh Dehghannasiri, Adam Gayoso, Alejandro A. Granados, Qiqing Li, Gita Mahmoudabadi, Aaron McGeever, Julia Eve Olivieri, Madeline Park, Neha Ravikumar, Geoff Stanley, Weilun Tan, Alexander J. Tarashansky, Rohan Vanheusden, Peter Wang, Sheng Wang, Galen Xing, Chenling Xu, Nir Yosef, Rebecca Culver, Les Dethlefsen, Po-Yi Ho, Shixuan Liu, Jonathan S. Maltzman, Ross J. Metzger, Koki Sasagawa, Rahul Sinha, Hanbing Song, Bruce Wang, Steven E. Artandi, Philip A. Beachy, Michael F. Clarke, Linda C. Giudice, Franklin W. Huang, Kerwyn Casey Huang, Juliana Idoyaga, Seung K. Kim, Christin S. Kuo, Patricia Nguyen, Thomas A. Rando, Kristy Red-Horse, Jeremy Reiter, David A. Relman, Justin L. Sonnenburg, Albert Wu, Sean M. Wu, Tony Wyss-Coray, Stephen R. Quake

**Affiliations:** 1 Department of Chemical Engineering, Stanford University, Stanford, CA USA; 2 ChEM-H, Stanford University, Stanford, CA USA; 3 Department of Bioengineering, Stanford University, Stanford, CA USA; 4 Department of Applied Physics, Stanford University, Stanford, CA USA; 5 Chan Zuckerberg Biohub, San Francisco, CA USA; 6 Chan Zuckerberg Biohub, San Francisco, CA USA; 7 Department of Biochemistry, Stanford University School of Medicine, Stanford, CA USA; 8 Howard Hughes Medical Institute, San Francisco CA, USA; 9 Department of Biomedical Data Science, Stanford University, Stanford, CA USA; 10 Center for Computational Biology, University of California, Berkeley, Berkeley, CA USA; 11 Department of Electrical Engineering and Computer Sciences, University of California, Berkeley, Berkeley, CA USA; 12 Ragon Institute of MGH, MIT and Harvard, Cambridge, MA USA; 13 Donor Network West, San Ramon, CA USA; 14 Department of Otolaryngology-Head and Neck Surgery, Stanford University School of Medicine, Stanford, CA USA; 15 Department of Surgery, University of California, San Francisco, San Francisco, CA USA; 16 Diabetes Center, University of California, San Francisco, San Francisco, CA USA; 17 DCI Donor Services, Sacramento, CA USA; 18 Department of Pathology, Stanford University School of Medicine, Stanford, CA USA; 19 Department of Microbiology and Immunology, Stanford University School of Medicine, Stanford, CA USA; 20 Department of Pediatrics, Division of Pulmonary Medicine, Stanford University, Stanford, CA USA; 21 Institute for Stem Cell Biology and Regenerative Medicine, Stanford University School of Medicine, Stanford, CA USA; 22 Department of Medicine, Division of Cardiovascular Medicine, Stanford University, Stanford, CA USA; 23 Department of Medicine and Liver Center, University of California, San Francisco, San Francisco, CA USA; 24 Department of Neurology and Neurological Sciences, Stanford University School of Medicine, Stanford, CA USA; 25 Department of Surgery - Plastic and Reconstructive Surgery, Stanford University School of Medicine, Stanford, CA USA; 26 Department of Developmental Biology, Stanford University School of Medicine, Stanford, CA USA; 27 Division of Infectious Diseases & Geographic Medicine, Department of Medicine, Stanford University, School of Medicine, Stanford, CA USA; 28 Department of Genetics, Stanford University School of Medicine, Stanford, CA USA; 29 Department of Biology, Stanford University, Stanford, CA USA; 30 Department of Pediatrics, Division of Cardiology, Stanford University School of Medicine, Stanford, CA USA; 31 Stanford Diabetes Research Center, Stanford University School of Medicine, Stanford, CA USA; 32 Center for Gynecology and Reproductive Sciences, Department of Obstetrics, Gynecology and, Reproductive Sciences, University of California, San Francisco, San Francisco, CA USA; 33 Department of Critical Care Medicine, The First Affiliated Hospital of Shantou University Medical College, Shantou, China; 34 Department of Ophthalmology, Stanford University School of Medicine, Stanford, CA USA; 35 Division of Nephrology, Department of Medicine, University of California, San Francisco, San Francisco, CA USA; 36 Stanford University School of Medicine, Stanford, CA USA; 37 Mass Spectrometry Platform, Chan Zuckerberg Biohub, Stanford, CA USA; 38 Stanford Cancer Institute, Stanford University School of Medicine, Stanford, CA USA; 39 Department of Medicine, Division of Hematology, Stanford University School of Medicine, Stanford, CA USA; 40 Department of Biochemistry and Biophysics, Cardiovascular Research Institute, University of California, San Francisco, San Francisco, CA USA; 41 Division of Hematology and Oncology, Department of Medicine, Bakar Computational Health Sciences Institute, Institute for Human Genetics, University of California, San Francisco, San Francisco, CA USA; 42 Stanford Cardiovascular Institute, Stanford, CA USA; 43 Department of Chemical and Systems Biology, Stanford University School of Medicine, Stanford, CA USA; 44 Department of Cell & Tissue Biology, University of California, San Francisco, San Francisco, CA USA; 45 Institute for Computational and Mathematical Engineering, Stanford University, Stanford, CA USA; 46 Paul F. Glenn Center for the Biology of Aging, Stanford University School of Medicine, Stanford, CA USA; 47 Division of Nephrology, Stanford University School of Medicine, Stanford, CA USA; 48 Veterans Affairs Palo Alto Health Care System, Palo Alto, CA USA; 49 Vera Moulton Wall Center for Pulmonary and Vascular Disease, Stanford University School of Medicine, Stanford, CA USA; 50 Department of Urology, Stanford University School of Medicine, Stanford, CA USA; 51 Division of Hematology/Oncology, Department of Medicine, San Francisco Veterans Affairs Health Care System, San Francisco, CA USA; 52 Department of Biochemistry, University of California, San Francisco, San Francisco, CA USA

**Keywords:** Machine learning, Diagnostic markers, Chronic kidney disease, Systems analysis, Next-generation sequencing

Correction to: *Nature Biotechnology* 10.1038/s41587-021-01188-9, published online 7 February 2022.

In the version of this article initially published, there was an error in the affiliation listed for Tabula Sapiens Consortium collaborator Albert Wu. The correct affiliation is Department of Ophthalmology, Stanford University School of Medicine, Stanford, CA, USA. The error has been corrected in the HTML and PDF versions of the article.

