# Supplementary material for: Cell types of origin of the cell-free transcriptome
Source: Nat Biotechnol. 2022 Feb 7;40(6):855–61. doi: 10.1038/s41587-021-01188-9 (PMC9200634; doi:10.1038/s41587-021-01188-9)
Supplement: Supplementary file 1 — Supplementary Fig. 1 and Supplementary Notes 1 and 2 [file 41587_2021_1188_MOESM1_ESM.pdf]

nature portfolio

https://doi.org/10.1038/s41587-021-01188-9

Supplementary information

Cell types of origin of the cell-free transcriptome

In the format provided by the authors and unedited

Supplementary Information for Vorperian et al. 'Cell types of origin of the cell free transcriptome'

Table of Contents

Supplementary Figure 1……………….………………………...………………………………..2-7
Supplementary Note 1…..……………..…………………………..……………………..……....8
Supplementary Note 2…..…………..……………………………..…………………………...9
The Tabula Sapiens Consortium Author List………………………………..……..………………10-12
References…..…………………………………………………….………………………………….13

Supplementary Figure 1

a

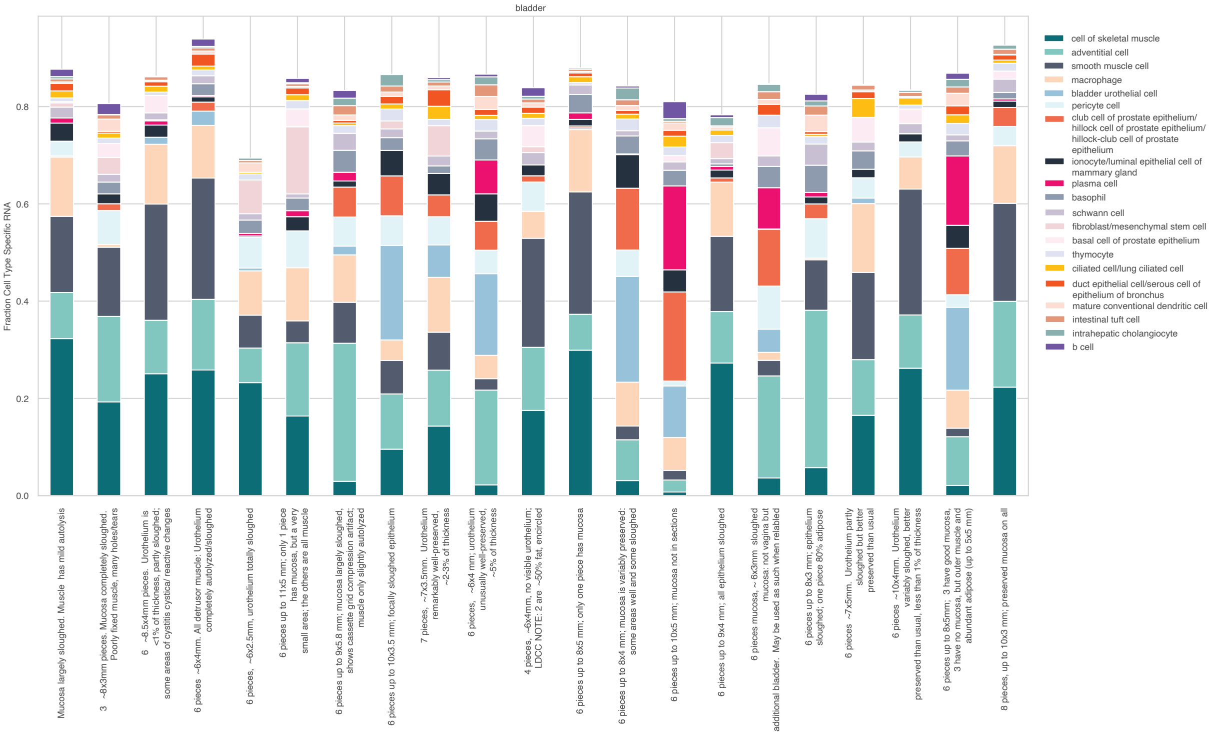

b

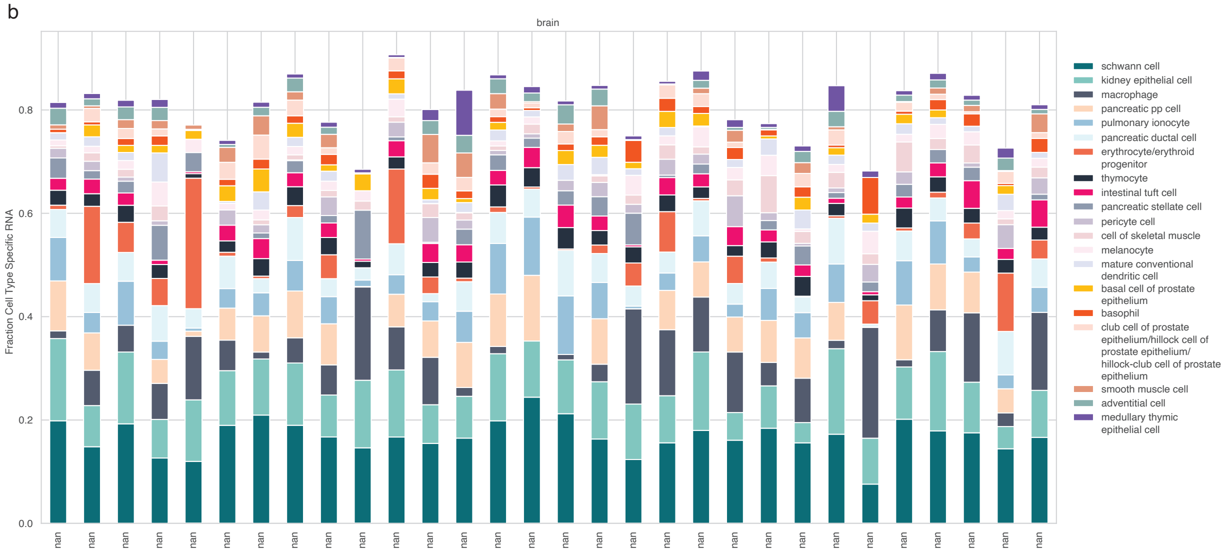

c

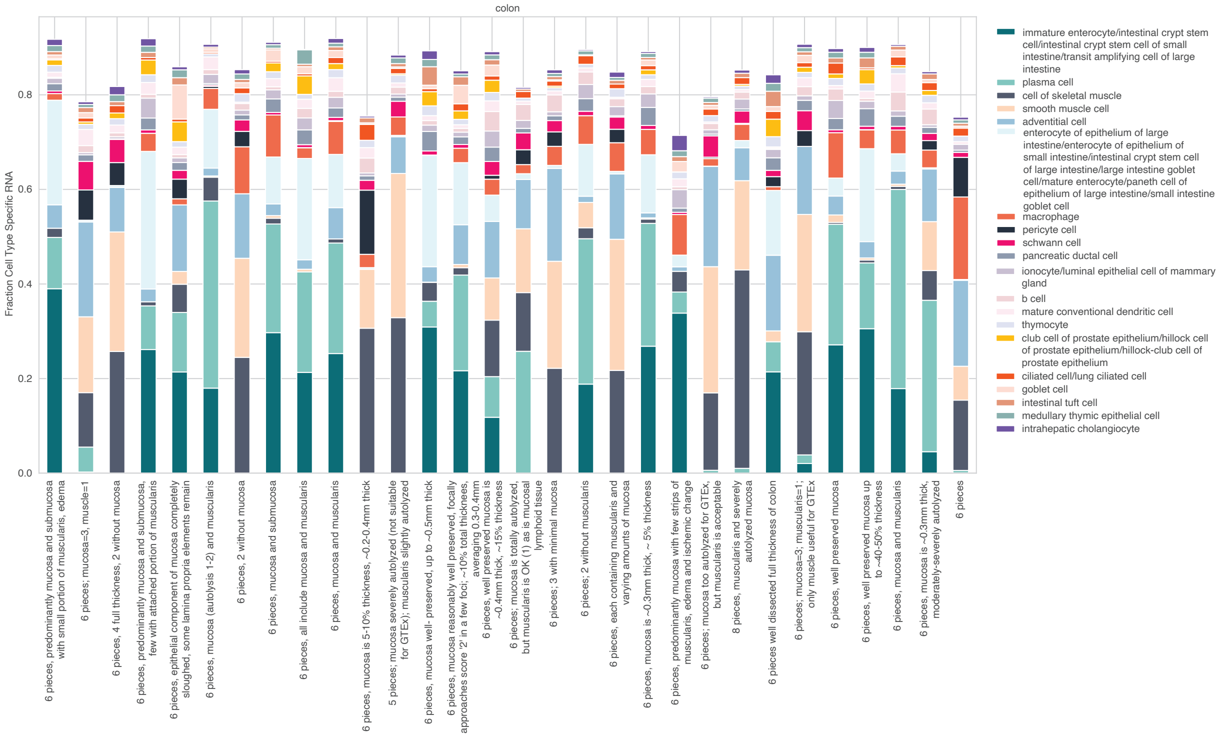
colon
Fraction Cell Type Specific RNA
0.0
0.2
0.4
0.6
0.8
6 pieces, predominantly mucosa and submucosa with small portion of muscularis, edema
6 pieces; mucosa=3, muscle=1
6 pieces, 4 full thickness, 2 without mucosa
6 pieces, predominantly mucosa and submucosa, few with attached portion of muscularis
6 pieces, epithelial component of mucosa completely sloughed, some lamina propria elements remain
6 pieces, mucosa (autolysis 1-2) and muscularis
6 pieces, 2 without mucosa
6 pieces, mucosa and submucosa
6 pieces, all include mucosa and muscularis
6 pieces, mucosa and muscularis
6 pieces, mucosa is 5-10% thickness, ~0.2-0.4mm thick
5 pieces; mucosa severely autolyzed (not suitable for GTEx); muscularis slightly autolyzed
6 pieces, mucosa well- preserved, up to ~0.5mm thick
6 pieces, mucosa reasonably well preserved, focally approaches score '2' in a few foci; ~10% total thickness, averaging 0.3-0.4mm
6 pieces, well preserved mucosa is ~0.4mm thick, ~15% thickness
6 pieces; mucosa is totally autolyzed, but muscularis is OK (1) as is mucosal lymphoid tissue
6 pieces; 3 with minimal mucosa
6 pieces; 2 without muscularis
6 pieces, each containing muscularis and varying amounts of mucosa
6 pieces, mucosa is ~0.3mm thick, ~ 5% thickness
6 pieces, predominantly mucosa with few strips of muscularis, edema and ischemic change
6 pieces; mucosa too autolyzed for GTEx, but muscularis is acceptable
8 pieces, muscularis and severely autolyzed mucosa
6 pieces well dissected full thickness of colon
6 pieces; mucosa=3; muscularis=1; only muscle useful for GTEx
6 pieces, well preserved mucosa
6 pieces, well preserved mucosa up to ~40-50% thickness
6 pieces, mucosa and muscularis
6 pieces, mucosa is ~0.3mm thick, moderately-severely autolyzed
6 pieces
immature enterocyte/intestinal crypt stem cell/intestinal crypt stem cell of small intestine/transit amplifying cell of large intestine
plasma cell
cell of skeletal muscle
smooth muscle cell
adventitial cell
enterocyte of epithelium of large intestine/enterocyte of epithelium of small intestine/intestinal crypt stem cell of large intestine/large intestine goblet cell/mature enterocyte/paneth cell of epithelium of large intestine/small intestine goblet cell
macrophage
pericyte cell
schwann cell
pancreatic ductal cell
ionocyte/luminal epithelial cell of mammary gland
b cell
mature conventional dendritic cell
thymocyte
club cell of prostate epithelium/hillock cell of prostate epithelium/hillock-club cell of prostate epithelium
ciliated cell/lung ciliated cell
goblet cell
intestinal tuft cell
medullary thymic epithelial cell
intrahepatic cholangiocyte

d

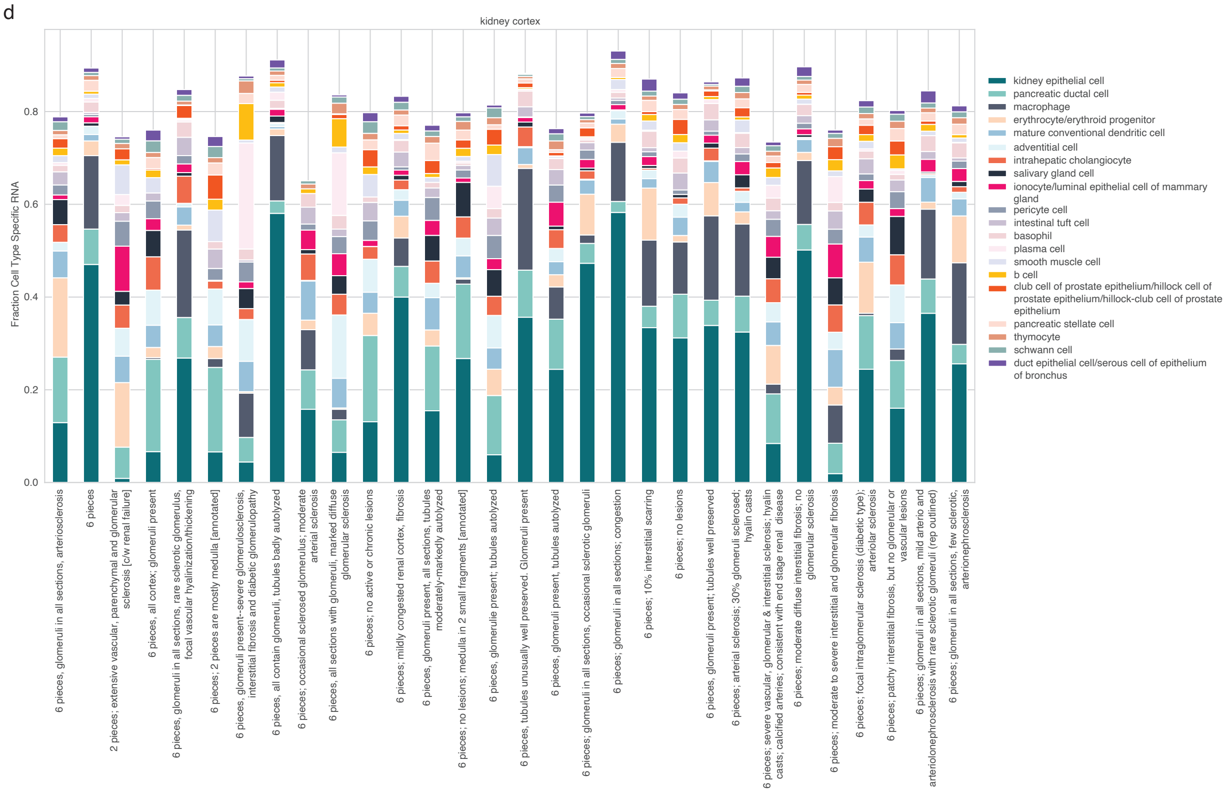
kidney cortex
Fraction Cell Type Specific RNA
0.0
0.2
0.4
0.6
0.8
6 pieces, glomeruli in all sections, arteriosclerosis
6 pieces
2 pieces; extensive vascular, parenchymal and glomerular sclerosis [c/w renal failure]
6 pieces, all cortex; glomeruli present
6 pieces, glomeruli in all sections, rare sclerotic glomerulus, focal vascular hyalinization/thickening
6 pieces; 2 pieces are mostly medulla [annotated]
6 pieces, glomeruli present--severe glomerulosclerosis, interstitial fibrosis and diabetic glomerulopathy
6 pieces, all contain glomeruli, tubules badly autolyzed
6 pieces; occasional sclerosed glomerulus; moderate arterial sclerosis
6 pieces, all sections with glomeruli, marked diffuse glomerular sclerosis
6 pieces; no active or chronic lesions
6 pieces; mildly congested renal cortex, fibrosis
6 pieces, glomeruli present, all sections, tubules moderately-markedly autolyzed
6 pieces; no lesions; medulla in 2 small fragments [annotated]
6 pieces, glomerulie present; tubules autolyzed
6 pieces, tubules unusually well preserved. Glomeruli present
6 pieces, glomeruli present, tubules autolyzed
6 pieces; glomeruli in all sections, occasional sclerotic glomeruli
6 pieces; glomeruli in all sections; congestion
6 pieces; 10% interstitial scarring
6 pieces; no lesions
6 pieces, glomeruli present; tubules well preserved
6 pieces; arterial sclerosis; 30% glomeruli sclerosed; hyalin casts
6 pieces; severe vascular, glomerular & interstitial sclerosis; hyalin casts; calcified arteries; consistent with end stage renal disease
6 pieces; moderate diffuse interstitial fibrosis; no glomerular sclerosis
6 pieces; moderate to severe interstitial and glomerular fibrosis
6 pieces; focal intraglomerular sclerosis (diabetic type); arteriolar sclerosis
6 pieces; patchy interstitial fibrosis, but no glomerular or vascular lesions
6 pieces; glomeruli in all sections, mild arterio and arterionephrosclerosis with rare sclerotic glomeruli (rep outlined)
6 pieces; glomeruli in all sections, few sclerotic, arterionephrosclerosis
kidney epithelial cell
pancreatic ductal cell
macrophage
erythrocyte/erythroid progenitor
mature conventional dendritic cell
adventitial cell
intrahepatic cholangiocyte
salivary gland cell
ionocyte/luminal epithelial cell of mammary gland
pericyte cell
intestinal tuft cell
basophil
plasma cell
smooth muscle cell
b cell
club cell of prostate epithelium/hillock cell of prostate epithelium/hillock-club cell of prostate epithelium
pancreatic stellate cell
thymocyte
schwann cell
duct epithelial cell/serous cell of epithelium of bronchus

e

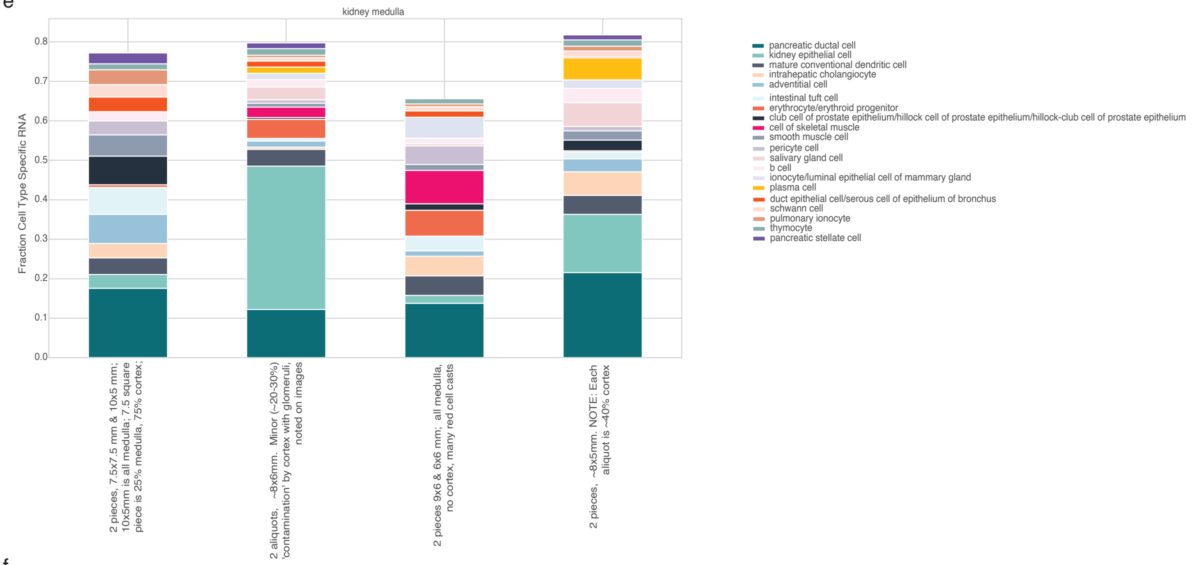
kidney medulla
Fraction Cell Type Specific RNA
0.0
0.1
0.2
0.3
0.4
0.5
0.6
0.7
0.8
2 pieces, 7.5x7.5 mm & 10x5 mm; 10x5mm is all medulla; 7.5 square piece is 25% medulla, 75% cortex;
2 aliquots, ~8x6mm. Minor (~20-30%) 'contamination' by cortex with glomeruli, noted on images
2 pieces 9x6 & 6x6 mm; all medulla, no cortex, many red cell casts
2 pieces, ~8x5mm. NOTE: Each aliquot is ~40% cortex
pancreatic ductal cell
kidney epithelial cell
mature conventional dendritic cell
intrahepatic cholangiocyte
adventitial cell
intestinal tuft cell
erythrocyte/erythroid progenitor
club cell of prostate epithelium/hillock cell of prostate epithelium/hillock-club cell of prostate epithelium
cell of skeletal muscle
smooth muscle cell
pericyte cell
salivary gland cell
b cell
ionocyte/luminal epithelial cell of mammary gland
plasma cell
duct epithelial cell/serous cell of epithelium of bronchus
schwann cell
pulmonary ionocyte
thymocyte
pancreatic stellate cell

f

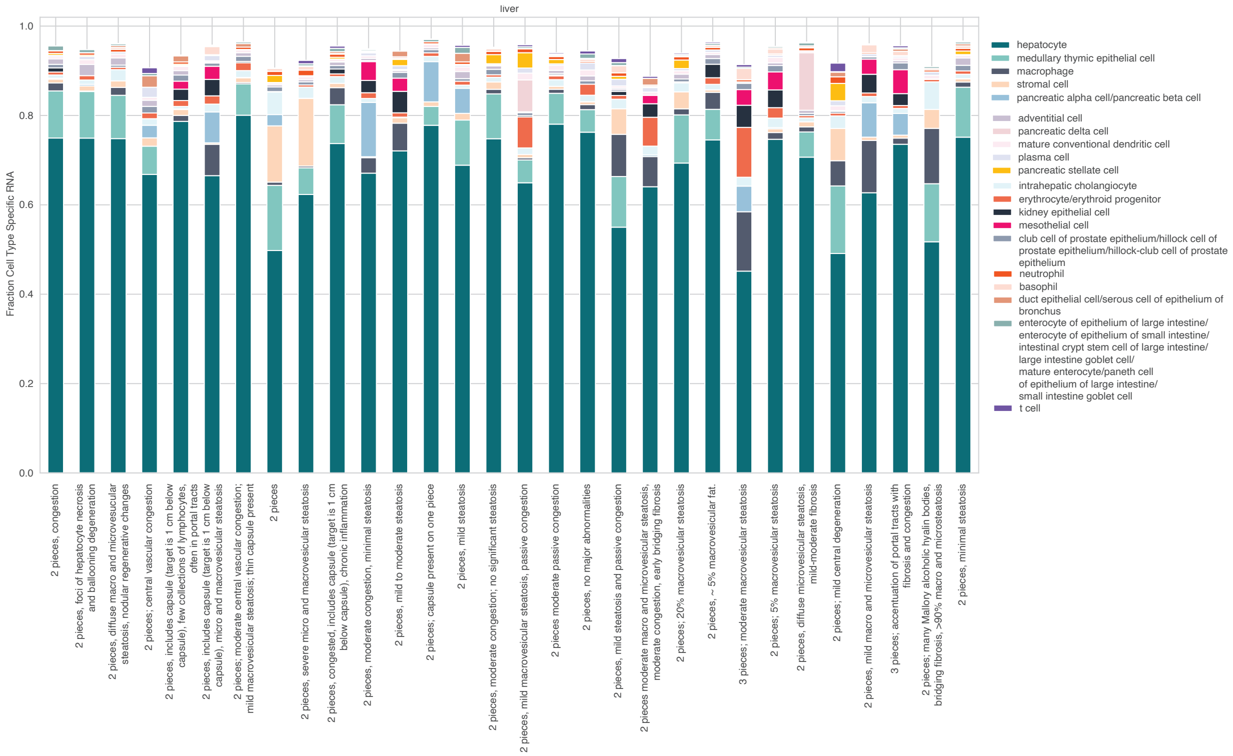
liver
Fraction Cell Type Specific RNA
0.0
0.2
0.4
0.6
0.8
1.0
2 pieces, congestion
2 pieces, foci of hepatocyte necrosis and ballooning degeneration
2 pieces, diffuse macro and microvesucular steatosis, nodular regenerative changes
2 pieces; central vascular congestion
2 pieces, includes capsule (target is 1 cm below capsule), few collections of lymphocytes, often in portal tracts
2 pieces, includes capsule (target is 1 cm below capsule), micro and macrovesicular steatosis
2 pieces; moderate central vascular congestion; mild macrovesicular steatosis; thin capsule present
2 pieces
2 pieces, severe micro and macrovesicular steatosis
2 pieces, congested, includes capsule (target is 1 cm below capsule), chronic inflammation
2 pieces, moderate congestion, minimal steatosis
2 pieces, mild to moderate steatosis
2 pieces; capsule present on one piece
2 pieces, mild steatosis
2 pieces, moderate congestion; no significant steatosis
2 pieces, mild macrovesicular steatosis, passive congestion
2 pieces moderate passive congestion
2 pieces, no major abnormalities
2 pieces, mild steatosis and passive congestion
2 pieces moderate macro and microvesicular steatosis, moderate congestion, early bridging fibrosis
2 pieces; 20% macrovesicular steatosis
2 pieces, ~ 5% macrovesicular fat.
3 pieces; moderate macrovesicular steatosis
2 pieces; 5% macrovesicular steatosis
2 pieces, diffuse microvesicular steatosis, mild-moderate fibrosis
2 pieces; mild central degeneration
2 pieces, mild macro and microvesicular steatosis
3 pieces; accentuation of portal tracts with fibrosis and congestion
2 pieces; many Mallory alcoholic hyalin bodies, bridging fibrosis, >90% macro and microsteatosis
2 pieces, minimal steatosis
hepatocyte
medullary thymic epithelial cell
macrophage
stromal cell
pancreatic alpha cell/pancreatic beta cell
adventitial cell
pancreatic delta cell
mature conventional dendritic cell
plasma cell
pancreatic stellate cell
intrahepatic cholangiocyte
erythrocyte/erythroid progenitor
kidney epithelial cell
mesothelial cell
club cell of prostate epithelium/hillock cell of prostate epithelium/hillock-club cell of prostate epithelium
neutrophil
basophil
duct epithelial cell/serous cell of epithelium of bronchus
enterocyte of epithelium of large intestine/enterocyte of epithelium of small intestine/intestinal crypt stem cell of large intestine/large intestine goblet cell/mature enterocyte/paneth cell of epithelium of large intestine/small intestine goblet cell
t cell

g

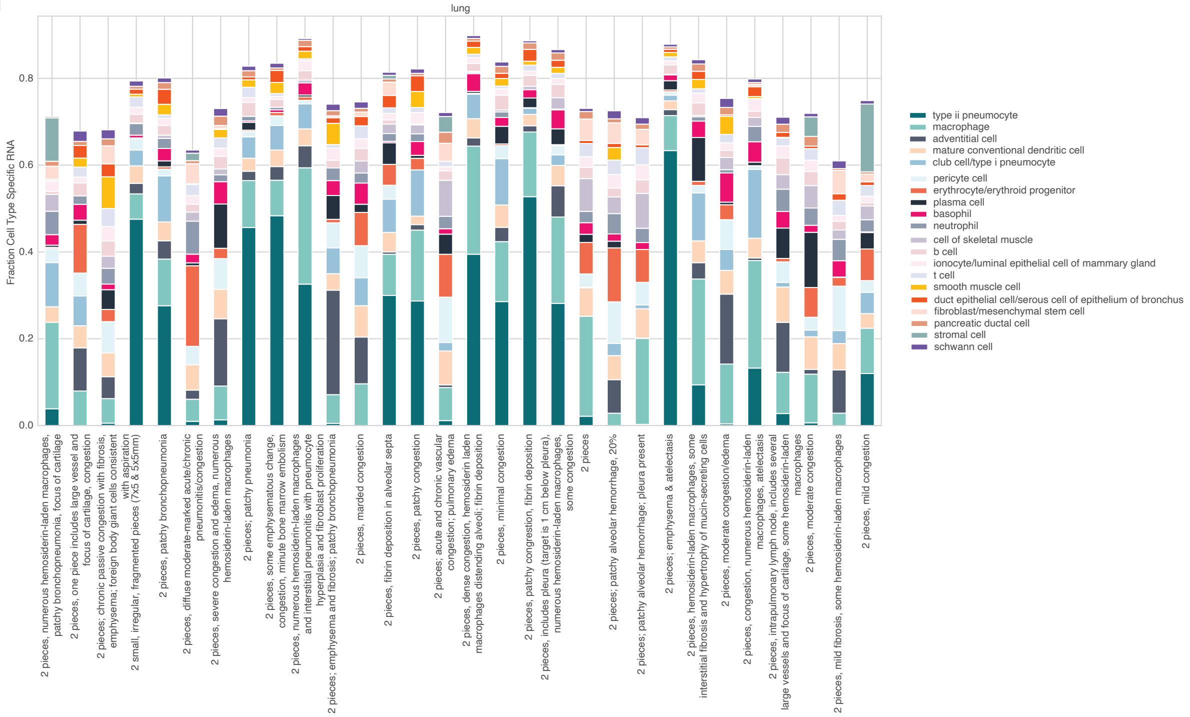
lung
Fraction Cell Type Specific RNA
type ii pneumocyte
macrophage
adventitial cell
mature conventional dendritic cell
club cell/type i pneumocyte
pericyte cell
erythrocyte/erythroid progenitor
plasma cell
basophil
neutrophil
cell of skeletal muscle
b cell
ionocyte/luminal epithelial cell of mammary gland
t cell
smooth muscle cell
duct epithelial cell/serous cell of epithelium of bronchus
fibroblast/mesenchymal stem cell
pancreatic ductal cell
stromal cell
schwann cell

h

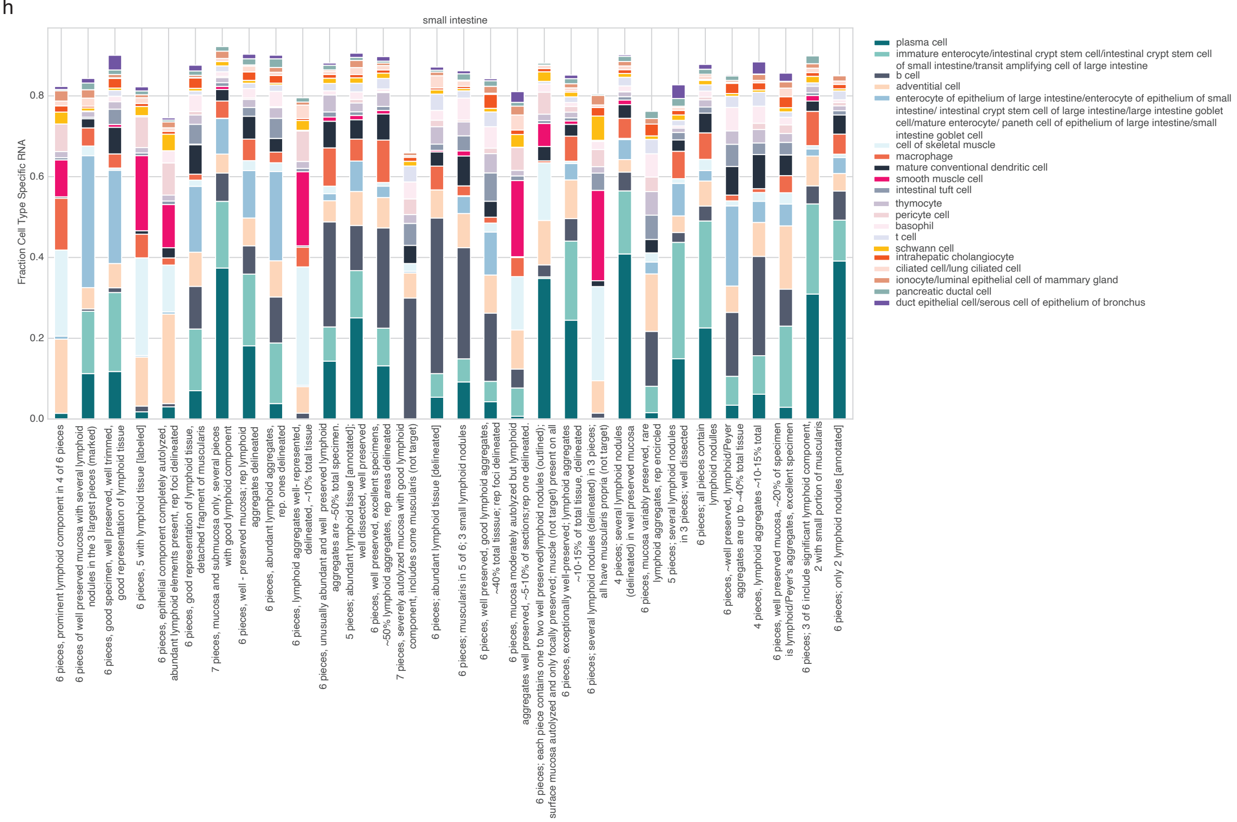
small intestine
Fraction Cell Type Specific RNA
plasma cell
immature enterocyte/intestinal crypt stem cell/intestinal crypt stem cell of small intestine/transit amplifying cell of large intestine
b cell
adventitial cell
enterocyte of epithelium of large intestine/enterocyte of epithelium of small intestine/ intestinal crypt stem cell of large intestine/large intestine goblet cell/mature enterocyte/ paneth cell of epithelium of large intestine/small intestine goblet cell
cell of skeletal muscle
macrophage
mature conventional dendritic cell
smooth muscle cell
intestinal tuft cell
thymocyte
pericyte cell
basophil
t cell
schwann cell
intrahepatic cholangiocyte
ciliated cell/lung ciliated cell
ionocyte/luminal epithelial cell of mammary gland
pancreatic ductal cell
duct epithelial cell/serous cell of epithelium of bronchus

i

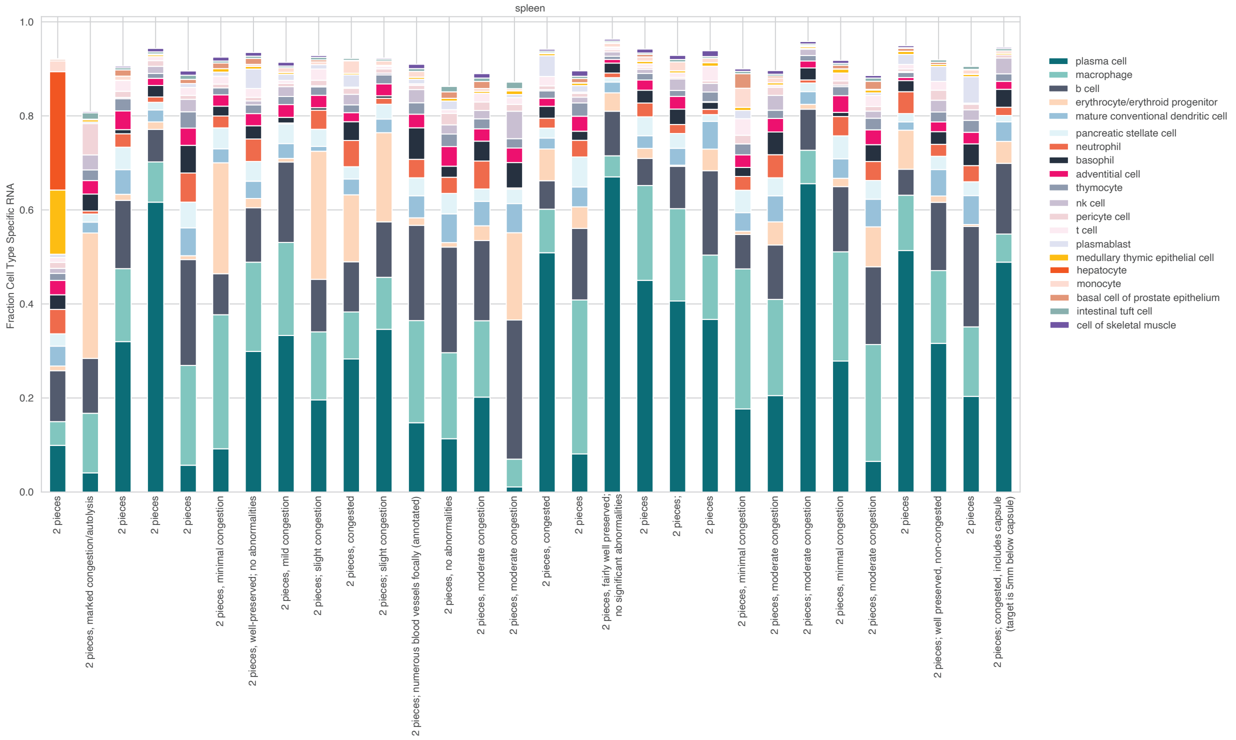
spleen
Fraction Cell Type Specific RNA
plasma cell
macrophage
b cell
erythrocyte/erythroid progenitor
mature conventional dendritic cell
pancreatic stellate cell
neutrophil
basophil
adventitial cell
thymocyte
nk cell
pericyte cell
t cell
plasmablast
medullary thymic epithelial cell
hepatocyte
monocyte
basal cell of prostate epithelium
intestinal tuft cell
cell of skeletal muscle

j

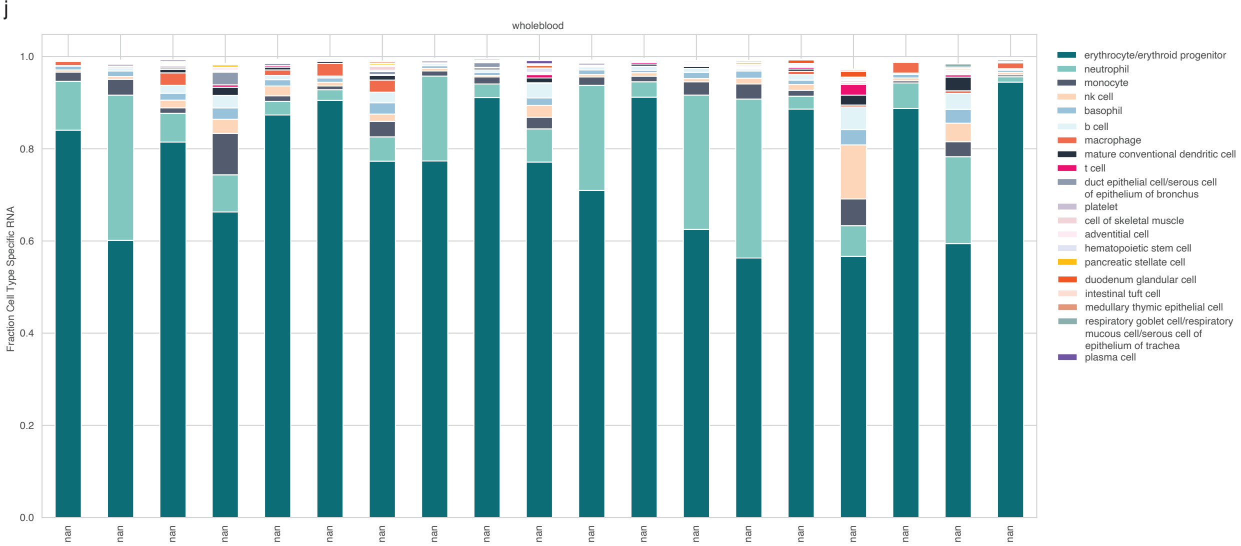
wholeblood
Fraction Cell Type Specific RNA
erythrocyte/erythroid progenitor
neutrophil
monocyte
nk cell
basophil
b cell
macrophage
mature conventional dendritic cell
t cell
duct epithelial cell/serous cell of epithelium of bronchus
platelet
cell of skeletal muscle
adventitial cell
hematopoietic stem cell
pancreatic stellate cell
duodenum glandular cell
intestinal tuft cell
medullary thymic epithelial cell
respiratory goblet cell/respiratory mucous cell/serous cell of epithelium of trachea
plasma cell

Supplementary Figure 1: Deconvolved fractions of cell type specific RNA from various GTEx tissues using nu-SVR and the *Tabula Sapiens* basis matrix.

Top 20 largest fractional contributions of cell type specific RNA for a given tissue. The two tissues whose cell types were absent from the basis matrix column space were Kidney – Medulla and Brain. Kidney medulla samples reported to be contaminated with cortex are reflected by deconvolved kidney epithelia fractions. The brain, which is absent from the TSP v1.0, yields majority fractions of schwann cell-specific RNA, a peripheral nervous cell type. Majority cell types for a given tissue, such as lung pneumonocytes and immune cells in the lung or kidney epithelia for the kidney cortex underscore the ability for the basis matrix to capture representative fractions of cell type specific RNA and reflect underlying cell heterogeneity in bulk RNA-seq data. Additional discussion is in Supplementary Note 1.

(a) Bladder
(b) Brain
(c) Colon – Transverse
(d) Kidney – Cortex
(e) Kidney – Medulla
(f) Liver
(g) Lung
(h) Small Intestine – Terminal Ileum
(i) Spleen
(j) Whole Blood

Supplementary Note 1: Deconvolution of bulk GTEx tissues using the *Tabula Sapiens*-derived basis matrix

To assess the ability of the basis matrix to deconvolve tissues whose cell types were wholly present in the cell type column space, we deconvolved a subset of bulk RNA-seq GTEx samples. The determined fractions of cell type specific RNA recapitulated the predominant cell types within a given tissue (Supplementary Information Fig. 1). Organs with increased cell type heterogeneity (lung, bladder, kidney, intestine, colon) in contrast to tissues with reduced spatial heterogeneity (liver, spleen, whole blood)[1], exhibited greater variance in deconvolved fractions (Supplementary Information Fig. 1) and deconvolution performance (Extended Data Fig. 3). Tissues with reduced spatial heterogeneity whose cell types were wholly in the basis matrix column space include predominantly b cells/plasma cells and erythrocytes in spleen; hepatocytes, liver; erythrocytes and leukocytes, whole blood. Cell types belonging to tissues with increased spatial heterogeneity exhibited greater variance in deconvolved fractions: kidney cortex majority fractions were from kidney epithelia and lymphocytes; small intestine, intestinal enterocytes and lymphocytes; lung, pneumonocytes and immune cells, colon, intestinal enterocytes, lymphocytes, and muscle cells. Cells with larger volume yielded larger deconvolved fractions across all tissues. Variance in the relative cell type fractional contributions across the deconvolved bulk samples within a given tissue reflects the underlying cell type heterogeneity, particularly in these complex samples. GTEx kidney medulla samples recorded to be contaminated with renal cortex reflect the presence of the kidney epithelia, the majority cell type in the renal cortex. Given that the kidney medulla is not part of TSP v1.0, we did not expect high deconvolution performance since its cell types are absent from the basis matrix column space. The brain, whose cell types were wholly absent from the cell type column space exhibited poor deconvolution performance, as expected. However, the majority cell type fraction assigned was to the cell type belonging to the peripheral nervous system that was present in Tabula Sapiens version 1, the schwann cell, underscoring the ability of our deconvolution method to assign fractional contributions to similar cell types from those that are absent from the basis matrix column space.

Supplementary Note 2: Noninvasive measurement of trophoblast cell type signatures in preeclampsia

In pregnancy, extravillous trophoblast (EVT) invasion is a stage in uteroplacental arterial remodeling[2,3]. Arterial remodeling occurs to ensure adequate maternal blood flow to the growing fetus[2,3] and is sometimes reduced in preeclampsia[2]. Previously, the EVT was reported by Tsang et al to be noninvasively resolvable and elevated in early onset preeclampsia (gestational age at diagnosis < 34 weeks) as compared to healthy pregnancy[4]. However, examination of the trophoblast gene profiles used by Tsang et al. using two independent placental single-cell atlases[3,5] revealed several genes that were not cell type specific or exhibited very low trophoblast expression (Extended Data Fig. 9c, d), thereby adversely impacting signature score interpretation.

CERCAM, *IL18BP*, and *PYCR1* are not extravillous trophoblast specific, exhibiting higher expression in fibroblast cell types in both atlases, despite Tsang's inclusion in their EVT gene profile (Extended Data Fig. 9c, d). Furthermore, EVT genes in Tsang's gene profile, *RRAD*, *SLC6A2*, and *UPK1B* all exhibit very low EVT expression across both placental atlases. Numerous PSG genes (*PSG11*, *PSG1/PSG2*, *PSG3*, *PSG4*, *PSG6*, *PSG9*) do not exhibit high syncytiotrophoblast (SCT) expression, despite their inclusion in Tsang's SCT gene profile. *GH2* either exhibits no expression or comparable non-SCT specific expression across cell types in both atlases (Extended Data Fig. 9c, d).

The presence of these non-cell type specific genes in a cell type gene profile consequently impacted the interpretation of Tsang et al's signature scores. Using our criteria for deriving a given cell type gene profile (Methods), we derived gene profiles for the same two cell types, EVT and SCT (Extended Data Fig. 8), and then quantified their respective signature scores in two previously published preeclampsia cohorts[6] (Extended Data Fig. 9a, b). In contrast to Tsang et al, we observed no significant difference in either trophoblast signature score in cfRNA samples collected at diagnosis for mothers with early-onset preeclampsia ($p = 0.703$ and $U = 1524$, 0.794 and $U = 1504$ respectively, two-sided Mann Whitney U) (Extended Data Fig. 9a) and for mothers with either early- or late-onset preeclampsia ($p = 0.24$ and $H = 4.18$, 0.54 and $H = 2.15$ respectively, Kruskal Wallace) (Extended Data Fig. 9b), as compared to samples from mothers with no complications at a matched gestational age.

In our work deriving cell type gene profiles for signature scoring in cfRNA, we only considered genes with high log fold change in a given cell type population and low expression in any other measured cell type (Methods). We acknowledge that this method may miss some genes for a given cell type population with low uniform expression (i.e. low expression in a large fraction of cells of a given type) or with heterogeneous expression (i.e. high expression in a small fraction of cells of a given type). However, since this work is the first comprehensive examination of cell type specific origins in the cell free transcriptome, we sought to be conservative in what we asserted to be cell type specific so that we could be confident in measuring a cell type signature score noninvasively; this approach boded well for all diseases presented in our work.

Taken together with validation in two independent placental cell atlases, we conclude that the EVT and SCT cell type gene profiles by Tsang et al. do not enable estimation of trophoblast pathology from cfRNA in preeclampsia. The role of extravillous trophoblast invasion and the ubiquity of its cellular pathophysiology in preeclampsia thus remains an open question.

The Tabula Sapiens Consortium Author List

Overall Project Direction and Coordination
Robert C. Jones[1], Jim Karkanias[2], Mark Krasnow[3,4], Angela Oliveira Pisco[2], Stephen R. Quake[1,2,5], Julia Salzman[3,6], Nir Yosef[2,7,8,9]

Donor Recruitment
Bryan Bulthaup[10], Phillip Brown[10], William Harper[10], Marisa Hemenez[10], Ravikumar Ponnusamy[10], Ahmad Salehi[10], Bhavani A. Sanagavarapu[10], Eileen Spallino[10]

Surgeons
Ksenia A. Aaron[11], Waldo Concepcion[10], James M. Gardner[12,13], Burnett Kelly[10,14], Nikole Neidlinger[10], Zifa Wang[10]

Logistical coordination
Sheela Crasta[1,2], Saroja Kolluru[1,2], Maurizio Morri[2], Angela Oliveira Pisco[2], Serena Y. Tan[15], Kyle J. Travaglini[3], Chenling Xu[7]

Organ Processing
Marcela Alcántara-Hernández[16], Nicole Almanzar[17], Jane Antony[18], Benjamin Beyersdorf[19], Deviana Burhan[20], Kruti Calcuttawala[21], Matthew M. Carter[16], Charles K. F. Chan[18,22], Charles A. Chang[23], Stephen Chang[3,19], Alex Colville[21,24], Sheela Crasta[1,2], Rebecca N. Culver[25], Ivana Cvijović[1,5], Gaetano D'Amato[26], Camille Ezran[3], Francisco X. Galdos[18], Astrid Gillich[3], William R. Goodyer[27], Yan Hang[23,28], Alyssa Hayashi[1], Sahar Houshdaran[29], Xianxi Huang[19,30], Juan C. Irwin[29], SoRi Jang[3], Julia Vallve Juanico[29], Aaron M. Kershner[18], Soochi Kim[21,24], Bernhard Kiss[18], Saroja Kolluru[1,2], William Kong[18], Maya E. Kumar[17], Angera H. Kuo[18], Rebecca Leylek[16], Baoxiang Li[31], Gabriel B. Loeb[32], Wan-Jin Lu[18], Sruthi Mantri[33], Maxim Markovic[1], Patrick L. McAlpine[11,34], Antoine de Morree[21,24], Maurizio Morri[2], Karim Mrouj[18], Shravani Mukherjee[31], Tyler Muser[17], Patrick Neuhöfer[3,35,36], Thi D. Nguyen[37], Kimberly Perez[16], Ragini Phansalkar[26], Angela Oliveira Pisco[2], Nazan Puluca[18] , Zhen Qi[18], Poorvi Rao[20], Hayley Raquer-McKay[16], Nicholas Schaum[18,21], Bronwyn Scott[31], Bobak Seddighzadeh[38], Joe Segal[20], Sushmita Sen[29], Shaheen Sikandar[18], Sean P. Spencer[16], Lea Steffes[17], Varun R. Subramaniam[31], Aditi Swarup[31], Michael Swift[1,] Kyle J. Travaglini[3], Will Van Treuren[16], Emily Trimm[26], Stefan Veizades[19,39], Sivakamasundari Vijayakumar[18], Kim Chi Vo[29], Sevahn K. Vorperian[1,40], Wanxin Wang[29], Hannah N.W. Weinstein[38], Juliane Winkler[41], Timothy T.H. Wu[3], Jamie Xie[38], Andrea R.Yung[3], Yue Zhang[3]

Sequencing
Angela M. Detweiler[2], Honey Mekonen[2], Norma F. Neff[2], Rene V. Sit[2], Michelle Tan[2], Jia Yan[2]

Histology
Gregory R. Bean[15], Vivek Charu[15], Erna Forgó[15], Brock A. Martin[15], Michael G. Ozawa[15], Oscar Silva[15], Serena Y. Tan[15], Angus Toland[15], Venkata N.P. Vemuri[2]

Data Analysis
Shaked Afik[7], Kyle Awayan[2], Olga Borisovna Botvinnik[2], Ashley Byrne[2], Michelle Chen[1], Roozbeh Dehghannasiri[3,6], Angela M. Detweiler[2], Adam Gayoso[7], Alejandro A Granados[2], Qiqing Li[2], Gita Mahmoudabadi[1], Aaron McGeever[2], Antoine de Morree[21,24], Julia Eve Olivieri[3,6,42], Madeline Park[2], Angela Oliveira Pisco[2], Neha Ravikumar[1], Julia Salzman[3,6], Geoff Stanley[1], Michael Swift[1], Michelle Tan[2], Weilun Tan[2], Alexander J Tarashansky[2], Rohan Vanheusden[2], Sevahn K. Vorperian[1,40], Peter Wang[3,6], Sheng Wang[2], Galen Xing[2], Chenling Xu[6], Nir Yosef[2,6,7,8]

Expert Cell Type Annotation
Marcela Alcántara-Hernández[16], Jane Antony[18], Charles K. F. Chan[18,22], Charles A. Chang[23], Alex Colville[21,24], Sheela Crasta[1,2], Rebecca Culver[25], Les Dethlefsen[43], Camille Ezran[3], Astrid Gillich[3], Yan Hang[23,28], Po-Yi Ho[16], Juan C. Irwin[29], SoRi Jang[3], Aaron M. Kershner[18], William Kong[18], Maya E Kumar[17], Angera H. Kuo[18], Rebecca Leylek[16], Shixuan Liu[3,44], Gabriel B. Loeb[32], Wan-Jin Lu[18], Jonathan S Maltzman[45,46], Ross J. Metzger[27,47], Antoine de Morree[21,24], Patrick Neuhöfer[3,35,36], Kimberly Perez[16], Ragini Phansalkar[26], Zhen Qi[18], Poorvi Rao[20], Hayley Raquer-McKay[16,] Koki Sasagawa[19,] Bronwyn Scott[31], Rahul Sinha[15,18,35], Hanbing Song[38], Sean P. Spencer[16], Aditi Swarup[31], Michael Swift[1], Kyle J. Travaglini[3], Emily Trimm[26], Stefan Veizades[19,39], Sivakamasundari Vijayakumar[18], Bruce Wang[20], Wanxin Wang[29], Juliane Winkler[41], Jamie Xie[38], Andrea R.Yung[3]

Tissue Expert Principal Investigators
Steven E. Artandi[3,35,36], Philip A. Beachy[18,23,48], Michael F. Clarke[18], Linda C. Giudice[29], Franklin W. Huang[38,49], Kerwyn Casey Huang[1,16], Juliana Idoyaga[16], Seung K Kim[23,28], Mark Krasnow[3,4], Christin S. Kuo[17], Patricia Nguyen[19,39,46], Stephen R. Quake[1,2,5], Thomas A. Rando[21,24], Kristy Red-Horse[26], Jeremy Reiter[50], David A. Relman[16,43,46], Justin L. Sonnenburg[16], Bruce Wang[20], Albert Wu[31], Sean M. Wu[19,39], Tony Wyss-Coray[21,24]

Affiliations
[1]Department of Bioengineering, Stanford University; Stanford, CA, USA.
[2]Chan Zuckerberg Biohub; San Francisco, CA, USA.
[3]Department of Biochemistry, Stanford University School of Medicine; Stanford, CA, USA.
[4]Howard Hughes Medical Institute; USA.
[5]Department of Applied Physics, Stanford University; Stanford, CA, USA.
[6]Department of Biomedical Data Science, Stanford University; Stanford, CA, USA.
[7]Center for Computational Biology, University of California Berkeley; Berkeley, CA, USA.
[8]Department of Electrical Engineering and Computer Sciences, University of California Berkeley; Berkeley, CA, USA.
[9]Ragon Institute of MGH, MIT and Harvard; Cambridge, MA, USA.
[10]Donor Network West; San Ramon, CA, USA.
[11]Department of Otolaryngology-Head and Neck Surgery, Stanford University School of Medicine; Stanford, California, USA.
[12]Department of Surgery, University of California San Francisco; San Francisco, CA, USA.
[13]Diabetes Center, University of California San Francisco; San Francisco, CA, USA.
[14]DCI Donor Services; Sacramento, CA, USA.
[15]Department of Pathology, Stanford University School of Medicine; Stanford, CA, USA.
[16]Department of Microbiology and Immunology, Stanford University School of Medicine; Stanford, CA, USA.
[17]Department of Pediatrics, Division of Pulmonary Medicine, Stanford University; Stanford, CA, USA.
[18]Institute for Stem Cell Biology and Regenerative Medicine, Stanford University School of Medicine; Stanford, CA, USA.
[19]Department of Medicine, Division of Cardiovascular Medicine, Stanford University; Stanford, CA, USA.
[20]Department of Medicine and Liver Center, University of California San Francisco; San Francisco, CA, USA.
[21]Department of Neurology and Neurological Sciences, Stanford University School of Medicine; Stanford, CA, USA.
[22]Department of Surgery - Plastic and Reconstructive Surgery, Stanford University School of Medicine; Stanford, CA, USA.
[23]Department of Developmental Biology, Stanford University School of Medicine, Stanford, CA, USA
[24]Paul F. Glenn Center for the Biology of Aging, Stanford University School of Medicine; Stanford, CA, USA.

[25]Department of Genetics, Stanford University School of Medicine; Stanford, CA, USA.
[26]Department of Biology, Stanford University; Stanford, CA, USA.
[27]Department of Pediatrics, Division of Cardiology, Stanford University School of Medicine; Stanford, CA, USA.
[28]Stanford Diabetes Research Center, Stanford University School of Medicine, Stanford, California
[29]Center for Gynecology and Reproductive Sciences, Department of Obstetrics, Gynecology and Reproductive Sciences, University of California San Francisco; San Francisco, CA, USA.
[30]Department of Critical Care Medicine, The First Affiliated Hospital of Shantou University Medical College; Shantou, China.
[31]Department of Ophthalmology, Stanford University School of Medicine; Stanford, CA, USA.
[32]Division of Nephrology, Department of Medicine, University of California San Francisco; San Francisco, CA, USA.
[33]Stanford University School of Medicine; Stanford, CA, USA.
[34]Mass Spectrometry Platform, Chan Zuckerberg Biohub; Stanford, CA, USA.
[35]Stanford Cancer Institute, Stanford University School of Medicine; Stanford, CA, USA.
[36]Department of Medicine, Division of Hematology, Stanford University School of Medicine, Stanford, CA, USA
[37]Department of Biochemistry and Biophysics, Cardiovascular Research Institute, University of California San Francisco; San Francisco, CA, USA.
[38]Division of Hematology and Oncology, Department of Medicine, Bakar Computational Health Sciences Institute, Institute for Human Genetics, University of California San Francisco; San Francisco, CA, USA.
[39]Stanford Cardiovascular Institute; Stanford CA, USA.
[40]Department of Chemical Engineering, Stanford University; Stanford, CA, USA.
[41]Department of Cell & Tissue Biology, University of California San Francisco; San Francisco, CA, USA.
[42]Institute for Computational and Mathematical Engineering, Stanford University; Stanford, CA, USA.
[43]Division of Infectious Diseases & Geographic Medicine, Department of Medicine, Stanford University School of Medicine; Stanford, CA, USA.
[44]Department of Chemical and Systems Biology, Stanford University School of Medicine, Stanford, CA, USA
[45]Division of Nephrology, Stanford University School of Medicine; Stanford, CA, USA.
[46]Veterans Affairs Palo Alto Health Care System; Palo Alto, CA, USA.
[47]Vera Moulton Wall Center for Pulmonary and Vascular Disease, Stanford University School of Medicine; Stanford, CA, USA.
[48]Department of Urology, Stanford University School of Medicine, Stanford, CA, USA
[49]Division of Hematology/Oncology, Department of Medicine, San Francisco Veterans Affairs Health Care System, San Francisco, CA, USA.
[50]Department of Biochemistry, University of California San Francisco; San Francisco, CA, USA.

References

1. The Tabula Sapiens Consortium & Quake, S. R. The Tabula Sapiens: a single cell transcriptomic atlas of multiple organs from individual human donors. *BioRxiv* (2021) doi:10.1101/2021.07.19.452956.

2. Kaufmann, P., Black, S. & Huppertz, B. Endovascular trophoblast invasion: implications for the pathogenesis of intrauterine growth retardation and preeclampsia. *Biol. Reprod.* **69**, 1–7 (2003).

3. Vento-Tormo, R. *et al.* Single-cell reconstruction of the early maternal-fetal interface in humans. *Nature* **563**, 347–353 (2018).

4. Tsang, J. C. H. *et al.* Integrative single-cell and cell-free plasma RNA transcriptomics elucidates placental cellular dynamics. *Proc Natl Acad Sci USA* **114**, E7786–E7795 (2017).

5. Suryawanshi, H. *et al.* A single-cell survey of the human first-trimester placenta and decidua. *Sci. Adv.* **4**, eaau4788 (2018).

6. Munchel, S. *et al.* Circulating transcripts in maternal blood reflect a molecular signature of early-onset preeclampsia. *Sci. Transl. Med.* **12**, (2020).
