# Supplementary material for: Cell types of origin of the cell-free transcriptome
Source: Nat Biotechnol. 2022 Feb 7;40(6):855–61. doi: 10.1038/s41587-021-01188-9 (PMC9200634; doi:10.1038/s41587-021-01188-9)
Supplement: Supplementary file 2 — Reporting Summary [file 41587_2021_1188_MOESM2_ESM.pdf]

nature research

Corresponding author(s): Dr. Stephen R. Quake

Last updated by author(s): November 21, 2021

Reporting Summary

Nature Research wishes to improve the reproducibility of the work that we publish. This form provides structure for consistency and transparency in reporting. For further information on Nature Research policies, see our Editorial Policies and the Editorial Policy Checklist.

Statistics

For all statistical analyses, confirm that the following items are present in the figure legend, table legend, main text, or Methods section.

n/a	Confirmed	
☐	☒	The exact sample size (*n*) for each experimental group/condition, given as a discrete number and unit of measurement
☐	☒	A statement on whether measurements were taken from distinct samples or whether the same sample was measured repeatedly
☐	☒	The statistical test(s) used AND whether they are one- or two-sided<br>*Only common tests should be described solely by name; describe more complex techniques in the Methods section.*
☒	☐	A description of all covariates tested
☐	☒	A description of any assumptions or corrections, such as tests of normality and adjustment for multiple comparisons
☐	☒	A full description of the statistical parameters including central tendency (e.g. means) or other basic estimates (e.g. regression coefficient) AND variation (e.g. standard deviation) or associated estimates of uncertainty (e.g. confidence intervals)
☐	☒	For null hypothesis testing, the test statistic (e.g. *F*, *t*, *r*) with confidence intervals, effect sizes, degrees of freedom and *P* value noted<br>*Give P values as exact values whenever suitable.*
☒	☐	For Bayesian analysis, information on the choice of priors and Markov chain Monte Carlo settings
☒	☐	For hierarchical and complex designs, identification of the appropriate level for tests and full reporting of outcomes
☐	☒	Estimates of effect sizes (e.g. Cohen's *d*, Pearson's *r*), indicating how they were calculated

Our web collection on statistics for biologists contains articles on many of the points above.

Software and code

Policy information about availability of computer code

Data collection	No data were collected in this study; all data used were from other studies.
Data analysis	All analyses were performed using Python (version 3.6) and R (version 3.6.1)<br>Deconvolution was coded using scikitlearn (version 0.23.2)<br>Bioinformatic processing: STAR (version 2.7.3a), GATK (version 4.1.1), htseq-count (version 0.11.1), FastQC (v ersion0.11.8), snakemake (version 5.8.1), MultiQC (version 1.7).<br>Data structures: AnnData (version 0.7.4). Single cell objects received from authors as Seurat objects were converted to an intermediate loom file, loom files were read into python using loompy (version 3.0.6).<br>Statistics: scipy (version 1.5.1)<br>Single cell analysis: scanpy (version 1.6.0), Seurat (version 3.1.5)<br>Basis matrix was generated using CIBERSORTx (cibersortx.stanford.edu)<br>Normalization: in addition to built-in functions in scanpy, edgeR (version 3.28.1) for TMM normalization

For manuscripts utilizing custom algorithms or software that are central to the research but not yet described in published literature, software must be made available to editors and reviewers. We strongly encourage code deposition in a community repository (e.g. GitHub). See the Nature Research guidelines for submitting code & software for further information.

Data

Policy information about availability of data

All manuscripts must include a data availability statement. This statement should provide the following information, where applicable:

- Accession codes, unique identifiers, or web links for publicly available datasets
- A list of figures that have associated raw data
- A description of any restrictions on data availability

All datasets used for this work were publicly available, downloaded with permission, or directly requested from authors.
Cell free RNA: For samples from Ibarra et al (PRJNA517339), Toden et al (PRJNA574438), and Chalasani et al (PRJNA701722), raw sequencing data was obtained from the SRA with the respective accession number. Reads were mapped to the reference human genome (hg38). For samples from Munchel et al, processed counts tables were directly downloaded.

Tissue gene lists and NX counts were downloaded from HPA (www.proteinatlas.org, v19). GTEx raw expression was downloaded from the GTEx portal (https://www.gtexportal.org/home/datasets, GTEx analysis V8). Tabula Sapiens single cell data were received from the CZ-Biohub (https://tabula-sapiens-portal.ds.czbiohub.org, version 1.0). The brain single cell data were downloaded with permission from Synapse (https://www.synapse.org/#!Synapse:syn18485175) and associated ROSMAP metadata were downloaded with permission from Synapse (https://www.synapse.org/#!Synapse:syn3157322). The liver Seurat object was requested from Aizarani et al. For the placenta atlases, a Seurat object was requested from Suryawanshi et al and AnnData requested from Vento-Tormo et al. Kidney AnnData was downloaded (https://www.kidneycellatlas.org, Mature Full dataset).

Field-specific reporting

Please select the one below that is the best fit for your research. If you are not sure, read the appropriate sections before making your selection.

☒ Life sciences ☐ Behavioural & social sciences ☐ Ecological, evolutionary & environmental sciences

For a reference copy of the document with all sections, see nature.com/documents/nr-reporting-summary-flat.pdf

Life sciences study design

All studies must disclose on these points even when the disclosure is negative.

Sample size

Cell free RNA samples: the datasets involved in this study were selected on the basis of availability, size, and high data quality. All datasets that we had access to meeting these three criteria were used in this work. No sample size calculation was performed; all samples used in this work were from published peer-reviewed studies. The entirety of the published samples passing QC were used in this work.

Data exclusions

Cell free RNA samples: we estimated the 3' bias ratio, ribosomal fraction, and the ratio of the number of reads that mapped to intronic as compared to exonic regions of the genome. A sample with a value greater than previously published thresholds for any of these three metrics was excluded from subsequent analysis.

Single cell: a list of disassociation genes were eliminated prior to downstream analysis (e.g. differential expression) while working with the Tabula Sapiens data given that observed disassociation artifact in single cell data.

Replication

The cell free transcriptome in human health:
We used several independent methods to assess the presence of cell-type specific signal, using cell type markers from PanglaoDB, systems-level deconvolution using Tabula Sapiens, and then individual cell type signatures scores derived from independent scRNA-seq tissue cell atlases. For systems level deconvolution on 75 healthy plasma samples, concordance was observed between the coefficients of cell type specific RNA between independent biological replicates between four different sample centers. For signature scoring and the cell type markers analyses, findings were again upheld over independent biological replicates.

The cell free transcriptome in pathology:
For the preeclampsia cell type signature scoring, we performed signature scoring using two independent datasets (PEARL-PEC and iPEC, from Munchel et al.). We validated our placental cell type signatures using two independent placental cell atlases (Munchel et al + Suryawanshi et al).

All cell type signature scores were tested between control and sick samples with a Mann-Whitney U test. We ensured that the resulting p-values were calibrated with a permutation test. Here, the labels compared in a given test (i.e. CKD vs. CTRL, AD vs. NCI, NAFLD vs. CTRL, etc.) were randomly shuffled 10,000 times. We observed a well-calibrated, uniform p-value distribution, validating the experimentally observed test statistics.

Of the differentially expressed genes that we observed to be cell type specific in AD/NAFLD, we performed a 10,000 trial permutation test on the Gini coefficients that are tissue-specific (e.g. brain/liver) vs. cell type specific. We found that the DEG that were identified as cell type specific possessed higher Gini than just tissue-specific. Together, this underscored that a subset of the DEG in cfRNA liquid biopsy for AD/NAFLD are associated with pathologically implicated cell types and are resolvable at cell type resolution.

All attempts at replication were successful.

Randomization

Randomization was not relevant for this study. For the determination of the healthy cf-transcriptome landscape, we looked at the signal observed within a given sample independently, then compared the observed results between different patients. In disease, comparisons were made solely on the basis of patient disease status, no treatments were applied.

Blinding	All data used in this work were publicly available; no data collection was performed, hence no blinding was necessary. QC filtering of samples was performed blinded before revealing disease status of the samples. During data analysis, as purpose of this study was to determine the comprehensive landscape of cell type specific signal in cfRNA as a baseline from which to measure aberrations in Chronic Kidney Disease/ Preeclampsia/Alzheimer's Disease/NAFLD and NASH, blinding was not performed.

Reporting for specific materials, systems and methods

We require information from authors about some types of materials, experimental systems and methods used in many studies. Here, indicate whether each material, system or method listed is relevant to your study. If you are not sure if a list item applies to your research, read the appropriate section before selecting a response.

Materials & experimental systems

n/a	Involved in the study
☒	☐ Antibodies
☒	☐ Eukaryotic cell lines
☒	☐ Palaeontology and archaeology
☒	☐ Animals and other organisms
☒	☐ Human research participants
☒	☐ Clinical data
☒	☐ Dual use research of concern

Methods

n/a	Involved in the study
☒	☐ ChIP-seq
☒	☐ Flow cytometry
☒	☐ MRI-based neuroimaging
